# A Review of Cancer Genetics and Genomics Studies in Africa

**DOI:** 10.3389/fonc.2020.606400

**Published:** 2021-02-15

**Authors:** Solomon O. Rotimi, Oluwakemi A. Rotimi, Bodour Salhia

**Affiliations:** ^1^Department of Translational Genomics, Keck School of Medicine, University of Southern California, Los Angeles, CA, United States; ^2^Department of Biochemistry, Covenant University, Ota, Nigeria; ^3^Norris Comprehensive Cancer Centre, University of Southern California, Los Angeles, CA, United States

**Keywords:** cancer, genetics, genomics, Africa, molecular biology

## Abstract

Cancer is the second leading cause of death globally and is projected to overtake infectious disease as the leading cause of mortality in Africa within the next two decades. Cancer is a group of genomic diseases that presents with intra- and inter-population unique phenotypes, with Black populations having the burden of morbidity and mortality for most types. At large, the prevention and treatment of cancers have been propelled by the understanding of the genetic make-up of the disease of mostly non-African populations. By the same token, there is a wide knowledge gap in understanding the underlying genetic causes of, and genomic alterations associated with, cancer among black Africans. Accordingly, we performed a review of the literature to survey existing studies on cancer genetics/genomics and curated findings pertaining to publications across multiple cancer types conducted on African populations. We used PubMed MeSH terms to retrieve the relevant publications from 1990 to December 2019. The metadata of these publications were extracted using R text mining packages: RISmed and Pubmed.mineR. The data showed that only 0.329% of cancer publications globally were on Africa, and only 0.016% were on cancer genetics/genomics from Africa. Although the most prevalent cancers in Africa are cancers of the breast, cervix, uterus, and prostate, publications representing breast, colorectal, liver, and blood cancers were the most frequent in our review. The most frequently reported cancer genes were *BRCA1*, *BRCA2*, and *TP53*. Next, the genes reported in the reviewed publications’ abstracts were extracted and annotated into three gene ontology classes. Genes in the *cellular component* class were mostly associated with *cell part and organelle part*, while those in *biological process* and *molecular function* classes were mainly associated with cell process, biological regulation, and binding, and catalytic activity, respectively. Overall, this review highlights the paucity of research on cancer genomics on African populations, identified gaps, and discussed the need for concerted efforts to encourage more research on cancer genomics in Africa.

## Introduction

Cancer is the second leading cause of death globally (1). In Africa, cancer incidence and mortality continue to grow rapidly. According to the 2018 Globocan data, new cancer cases and cancer deaths in Africa were estimated at 1,049,800 and 700,800, respectively (2). In 2018, women in East Africa had the highest cumulative risk of dying from cancer globally. The burden of cancer in Africa is increasing, and this burden is expected to increase by 60% by the year 2030. To lower this projected increase in cancer burden, population-relevant biological studies and the identification of innate risk factors among African populations are needed (3–5).

As cancer is a genetic disease, scientific studies investigating its causes, diagnosis, and treatment in developing countries need to focus more on genetics and genomics. The African or Black population is not a homogenous group and, as such, necessitates the need for genomic/genetic studies to reflect the diverse African populations. The population history of Africa shows that the people of Africa are the most genetically and phenotypically diverse population (6, 7). The peopling history of Africa has been described by Campbell et al. and Tucci & Akey (8, 9), and their reviews showed that African ethnic groups and tribes are genetically heterogeneous. Hence, there is likely a critical contribution of the underlying within-group genetic differences to the disparity in cancer prognosis seen among Blacks (10). Therefore, cancer genetics/genomics studies are expected to significantly impact the understanding of the risk, susceptibility, diagnosis, and treatment of this disease.

The genomic heterogeneity of human populations was driven by ancient migration and heterogeneous adaptive pressures on the human genome, particularly on the African Continent (11, 12). These evolutionary events resulted in the split of human populations into five distinct groups: southern Khoe-San, northern Khoe-San, central African hunter-gatherers, West Africans, and East Africans, out of which a subset migrated out of Africa and is now recognized as the out-of-Africa population (11, 12). Therefore, the African continent could be considered to harbor the repository of human genomic diversity and serves as the resource reference for understanding the role of genomics in human health equity. This repository is further deepened by the present-day North African populations enriched with the genetic pool of the out-of-Africa’s Euro-Asian populations. Still, Africa’s contribution to global genetic and genomics information is grossly disproportionate to its population’s diversity and size. For example, very few African populations were included in the HapMap and 1000Genome projects (13). This is a serious shortcoming for a group of people that represent over 90% of human genomic diversity. A recent review of genome-wide association studies (GWAS) showed that Africans (including African Americans) only represent 2.4% of individuals included in all GWAS studies (14).

The proper understanding of genetics and genomics among African populations will expectantly improve prevention, diagnosis, and treatment outcomes of cancer. Although recent evidence shows that the burden of cancer is in Africa, there remains a huge deficit in requisite skills and infrastructure required to carry out the necessary research studies to alleviate this knowledge gap, requiring still non-African nations to fill this gap (15).

Accordingly, in this review, we discuss both genetics and genomics study findings across multiple cancer types in African populations. The goal here is to demonstrate the existing knowledge and to crucially identify the gaps that should be filled in order to address the cancer burden across Africa.

## Methods

**Figure 1:**
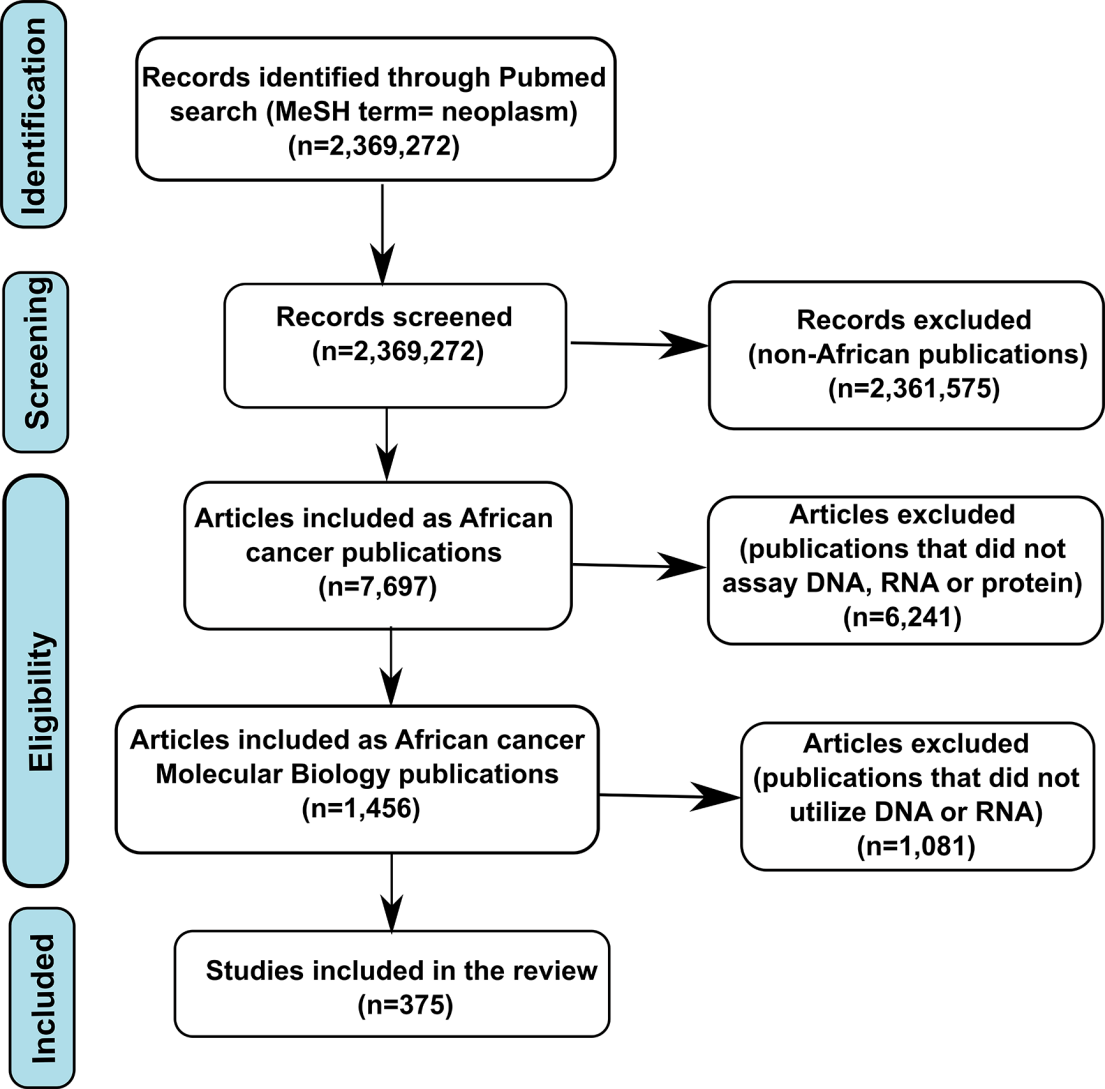
Flow diagram of the literature search strategy.

The peer-reviewed publications included in this review were extracted from PubMed and covered the period between January 1990 and December 2019, as shown in the flow chart in **Figure 1**. Since PubMed Medical Subject Heading (MeSH) terms involve synonym control, it yields more precise and inclusive search results (16). Our literature search approach, therefore, utilized an integration of MeSH terms that incorporated “the disease” (neoplasm), 54 African countries, and combinations of study parameters (‘gene or protein or molecular biology or mutation or genetics or genomics’). After extracting African cancer papers, we next filtered those to include only papers pertaining to cancer molecular biology (protein or nucleic acid). Cancer molecular biology papers were then further filtered using “genetic* OR genomic* OR mutation*[MeSH Terms].” The final criteria were that the studies must utilize biospecimens of African origin. Two authors (SOR and OAR) manually verified these publications to ensure the accuracy of terms.

For the purpose of data extraction, the metadata and abstract of each publication returned from our search were collected in a single corpus and subjected to text-mining using the R packages RISmed (17) and Pubmed.mineR (18). The publications returned were analyzed in R to identify the cancer types/sites associated with each publication, as described by Acharya et al. (19). Furthermore, the R package “PubmedmineR” was used for obtaining the names and frequency of occurrence of genes denoted in “Human Genome Nomenclature Committee” (HGNC) symbols (20). For this purpose, we considered the genes reported in the abstract as the genes associated with the most prominent findings of the publications. Next, these genes were pulled and subjected to gene ontology functional profiling for three gene ontology classes (“molecular function”, “biological process”, and “cellular component”) using “goProfiles” (21).

## Results

The total numbers of publications returned by our search on the topics of cancer globally, as well as cancer, cancer molecular biology, and cancer genetics/genomics within Africa between 1990 and December 2019, are shown in **Figure 1**. Out of nearly two and half million publications on cancer globally, only 7,697 (0.329%) papers were returned by our search on cancer in Africa, with only 1,456 (0.061%) related to molecular biology (protein or nucleic acid). Of these publications, only 375 articles were found using the search terms “genetic, genomics, mutations”.

**Figure 2:**
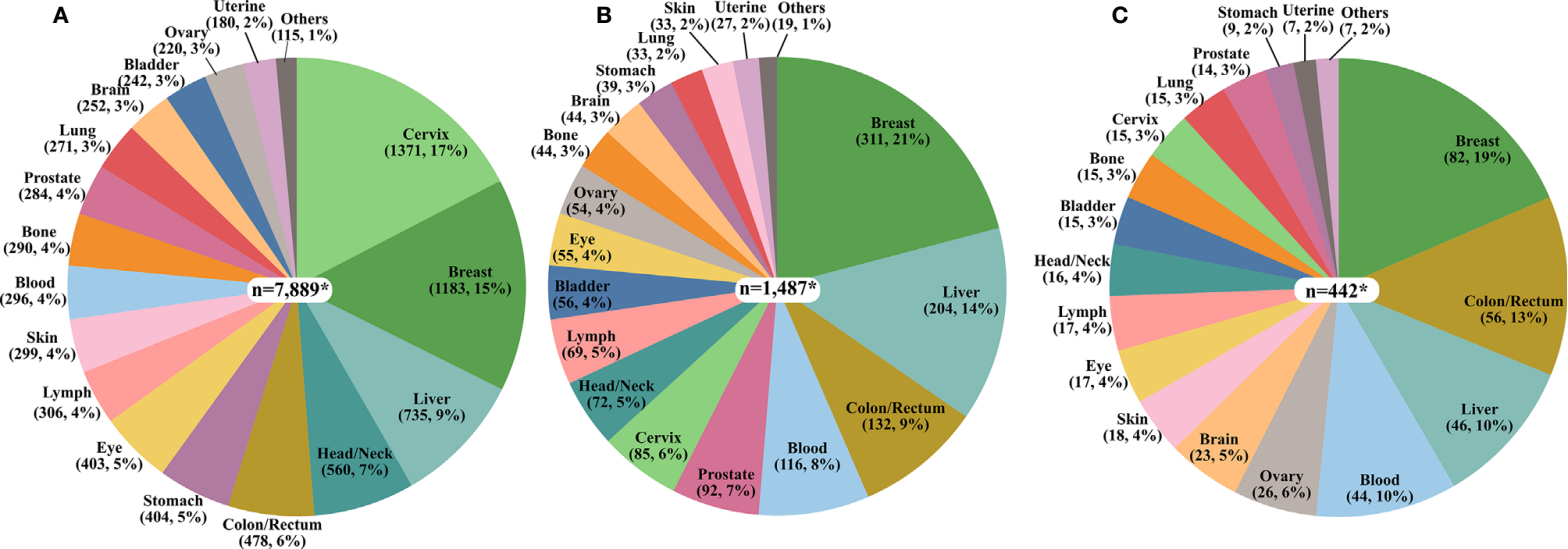
The proportion of the number of publications on each cancer type. **(A)** Cancer in Africa (n=7,697) **(B)** Cancer Molecular Biology in Africa (n=1,456), and **(C)** Cancer Genetics/Genomics in Africa (n=375). *The total values presented in the pie charts are greater than the sum of publications in each category due to the multiplicity of cancer sites for some publications as exemplified by studies on breast/ovary and blood/lymph.

Among all cancer publications pertaining to Africa, the cancer sites with the highest number of published studies represented cancers of the cervix, breast, liver, head/neck, and colorectal while, lung, brain, bladder, ovarian, and uterine cancers were the least frequently reported on (**Figure 2A**). For publications related to cancer molecular biology in Africa, breast, liver, colorectal, blood, and prostate cancer were the most frequent. In contrast, cancers of the brain, stomach, lung, skin, and uterine cancer had the fewest publications (**Figure 2B**). Most papers reporting cancer genetics or genomics reported on breast, colorectal, liver, blood, and ovarian cancer, with the fewest cancer genetics or genomics studies on the brain, stomach, lung, skin, and uterine cancers (**Figure 2C**).

**Figure 3:**
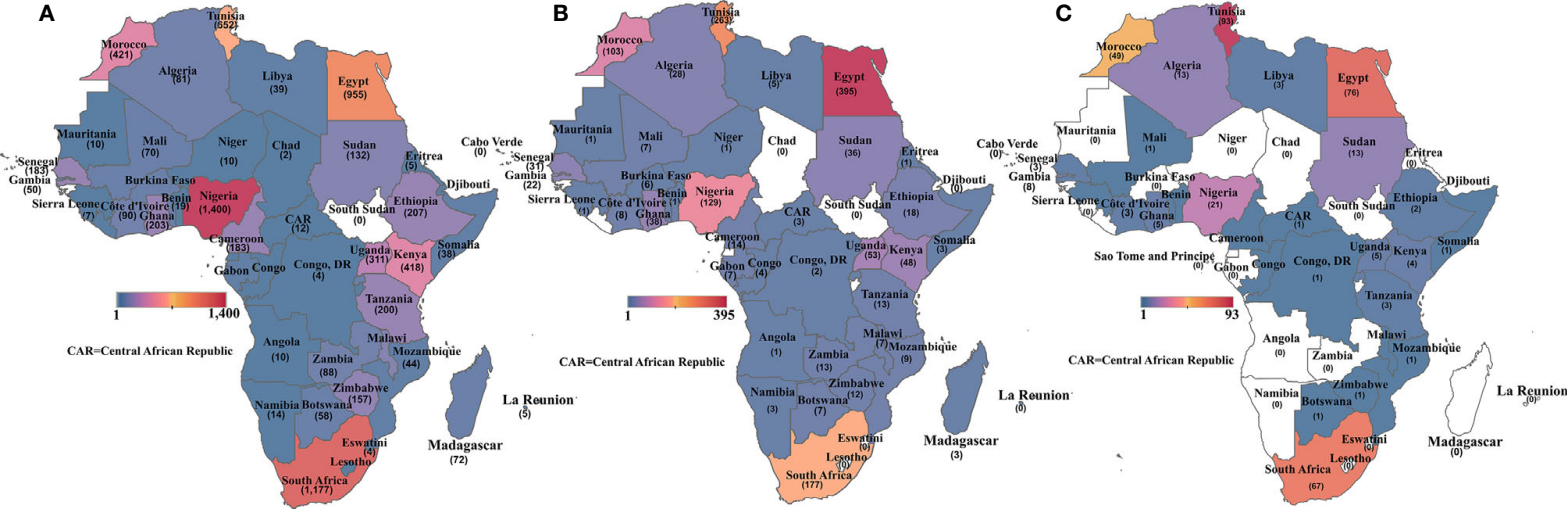
Heat map showing the number of publications retrieved for **(A)** all cancer publications per African country; **(B)** cancer molecular biology publications per African country and **(C)** cancer genetics/genomics publications per African country. Countries without any publication in each category are shaded in white.

There were also disparities in the publications by country, as illustrated in **Figures 3A–C**. Nigeria had the most papers on cancer overall, followed by South Africa, Egypt, Tunisia, Morocco, and Kenya (**Figure 3A**). For cancer molecular biology papers, Egypt took the lead, followed by Tunisia, South Africa, and then Nigeria (**Figure 3B**). Tunisia, however, returned the most search results for cancer genetics/genomics papers followed by Egypt, South Africa, and Morocco (**Figure 3C**). Overall, only seven African countries contributed at least 10 cancer genetics/genomics publications, while 22 African countries returned no search results on cancer genetics/genomics studies. The search results show clear evidence of regional differences in publishing capacity, with North Africa and South Africa leading in cancer research.

**Figure 4:**
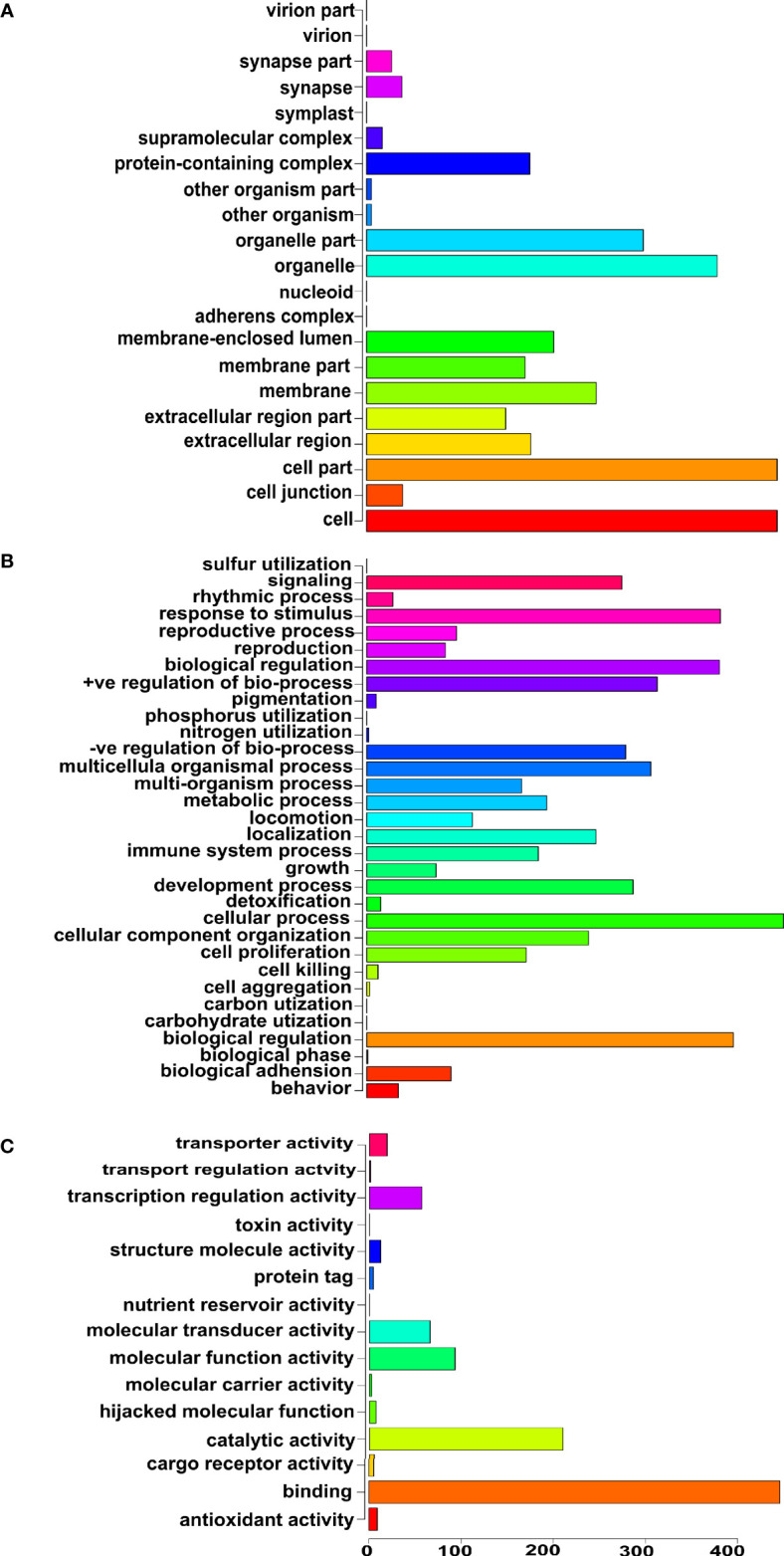
Gene ontology of the genes reported in the abstracts of publications on cancer genomics in Africa. **(A)** Cellular component ontology, **(B)** Biological process ontology, and **(C)** Molecular function ontology.

Next, we focused specifically on the list of 375 genetics/genomics publications for gene curation and review. We did this to identify the functional contributions of these studies to the understanding of biological processes associated with carcinogenesis, using functional correlations comparison (22). A total of 152 genes in the abstracts of 375 publications on cancer genomics were extracted and further annotated into the following gene ontology classes: *cellular component, biological process, and molecular function* (**Figures 4A–C**). In the *cellular component* class, the genes studied were mostly associated with cell part, organelle, organelle part, and cell membrane. In contrast, the genes in the biological process were mainly associated with cell process, biological regulation, response to stimulus, and positive regulation of the biological process. The *molecular function* ontology genes were mostly associated with binding, catalytic activity, molecular function regulator, molecular function transducer activity, and transcription regulation in the molecular function class, which are dysregulated in cancer. The most studied genes in the publications were *BRCA1*, *BRCA2*, *TP53*, *EGFR*, and *MLH1* (**Table 1**), indicating a dearth of data on the plethora of other critical cancer-associated genes. Next, we reviewed some of the key findings reported across the 375 genomics papers for each of the major and most frequently published cancer types below.

**Table 1:** List of top 20 genes reported in the abstracts of publications on cancer genetics and genomics in Africa.

	Gene symbol	Genes	Frequency
1	*BRCA1*	breast cancer 1, early onset	164
2	*BRCA2*	breast cancer 2, early onset	108
3	*TP53*	tumor protein p53	73
4	*EGFR*	epidermal growth factor receptor	53
5	*MLH1*	mutL homolog 1, colon cancer, nonpolyposis type 2 (E. coli)	41
6	*KRAS*	v-Ki-ras2 Kirsten rat sarcoma viral oncogene homolog	39
7	*BRAF*	v-raf murine sarcoma viral oncogene homolog B1	30
8	*XPA*	xeroderma pigmentosum, complementation group A	29
9	*RET*	ret proto-oncogene	22
10	*NPM1*	nucleophosmin (nucleolar phosphoprotein B23, numatrin)	21
11	*NLRP7*	NLR family, pyrin domain containing 7	20
12	*APC*	adenomatous polyposis coli	19
13	*JAK2*	Janus kinase 2	19
14	*MSH2*	mutS homolog 2, colon cancer, nonpolyposis type 1 (E. coli)	19
15	*ABCB1*	ATP-binding cassette, sub-family B (MDR/TAP), member 1	16
16	*GSTT1*	glutathione S-transferase theta 1	16
17	*MGMT*	O-6-methylguanine-DNA methyltransferase	15
18	*RB1*	retinoblastoma 1	15
19	*WT1*	Wilms tumor 1	15
20	*MDM2*	MDM2 oncogene, E3 ubiquitin protein ligase	14

### Breast/Ovarian Cancer

Breast cancer has continued to be the leading cause of cancer morbidity and mortality in Africa, with an incidence and mortality rate of 37.9 and 17.2 per 100000, respectively, according to GLOBOCAN 2018 data (2). Breast cancer’s prominence in Africa dates back to around 3000BC in the ancient Egyptian medical text - the Edwin Smith Papyrus, the oldest cancer record (23, 24). Not surprisingly, breast cancer had the highest number (n=82, 19%) of peer-reviewed cancer genetics/genomics publications in Africa. With the current understanding of cancer as a genomic disease and the unique phenotype that breast cancer presents in the people of African ancestry, attempts to address its burden require rigorous genomics investigations.

Together with cancer of the ovary, breast cancer risk is greatly increased in women with inherited mutation(s) in tumor suppressor genes (25). Not surprisingly, the earliest publications on breast and ovarian cancers in African populations focused on understanding the contribution of variations in the tumor suppressor genes *BRCA1/2* and *TP53*, particularly in North African populations of Morocco, Tunisia, Egypt, and Sudan (26–40). While these findings hold immense benefits for those populations, their *BRCA* variants are not dissimilar to those present in the out-of-Africa populations. This, therefore, limits the translational impact of such findings to controlling breast/ovarian cancer in the Sub-Saharan African populations.

Furthermore, the major epidemiological implication of *BRCA* mutations lies in identifying specific founder mutation(s) within each population, with the view of using it as a predictive molecular risk marker and treatment recommendation. For instance, advances in understanding the role of *BRCA* proteins in tumorigenesis have now led to improved therapeutic choices with the availability of PARP inhibitors for breast cancer patients with germline mutations (41). Also, the identification of founder *BRCA* gene mutations in populations like Ashkenazi-Jewish (Hungarian and Russian), Polish, Norwegian and Icelandic people has resulted in improved low-cost genetic testing and the determination of high-risk individuals for breast and ovarian cancers (42, 43). Therefore, these have made it imperative for the founder mutations of the *BRCA* gene within Africa populations to be identified and included in breast cancer screening, diagnosis, and treatment.

In an attempt to consider *BRCA* contributions to breast cancer in Africa, Rebbeck et al. (44) published a global distribution of *BRCA1* and *BRCA2* germline mutations by including women from Nigeria and South Africa. However, the extent to which their subjects represent the ethnic and genetic diversity in these countries is unclear. They did note that the mutations observed in African American families were of African origin because they are unlike the mutations seen in out-of-African ethnic groups (44–46). This study of Rebbeck et al. (44) was part of the Consortium of Investigators of Modifiers of *BRCA1/2* investigations, which only included the nation of South Africa (http://cimba.ccge.medschl.cam.ac.uk/cimba-groups/study-groups/). A more detailed study of Zheng et al. (47) on Nigerian women established that up to 20% of inherited invasive breast cancer cases in Nigeria are associated with inherited mutations in *BRCA1*, *BRCA2*, *PALB2*, or *TP53*. Their findings on *BRCA1* and *BRCA2* built on the earlier report of Fackenthal et al. (48) that Nigerian breast cancer patients have a very high frequency of *BRCA1* and *BRCA2* mutations. These mutations were reported by Pitt et al. (49) to be associated with greater structural variation and aggressive biology in Nigerian women with HR + /HER2 − tumors. Similar findings were reported by Pegoraro et al. (50) in Black South Africans with ovarian epithelial malignancies.

Recently, Mahfoudh et al. (51) showed that the 5382insC *BRCA1* mutation contributes to the development of triple-negative breast cancer (TNBC) in Tunisia. The higher mortality of breast cancer in women of West African ancestry is due in part to higher levels of TNBC (compared to whites), which is associated with the poorest prognosis of all breast cancer subtypes. Hence, *BRCA* screening in Africa could help identify women who can benefit from *PARP* inhibitors leading to improved clinical outcomes. In South Africa, Reeves et al. (52) characterized *BRCA1* mutations in breast and/or ovarian cancer to identify founder mutations in Afrikaner families. However, this population is also of European ancestry, and the mutations that were identified were similar to those reported in the Netherlands and in Ashkenazi Jews (53). They also reported that variants of *PALB2*, a partner and localizer of *BRCA2* was also associated with the early onset of breast cancer in some South African patients (53). *PALB2* functions as a scaffold between *BRCA1* and *BRCA2*. Similar *PALB2* mutations have previously been identified in women of European ancestry but not in women with Nigerian ancestry, as reported by Sluiter et al. (54).

The first publication on *BRCA* mutations in the indigenous Sub-Saharan African population was by Zhang et al. (55), who identified an ancient *BRCA1* mutation (Y101X) in Yoruba (Nigeria, West Africa) breast cancer patients. The team further reported a non-pathogenic novel exon 21 deletion of *BRCA1* (c. 5277 + 480_5332+672del) in Nigeria in addition to a novel deleterious *BRCA1* mutation (c. 1949_1950delTA) in a woman from Senegal (West Africa) (56). Another novel founder, *BRCA2* mutation, was identified by var der Merwe et al. (53) in the Bantu-speaking Xhosa population (South Africa). Other studies have identified new *BRCA* mutations and their contribution to early-onset and sporadic breast and/or ovarian cancer in Arabic speaking countries (57) of Egypt (58), Tunisia (51, 59–66), Algeria (67–69), Morocco (70–72), and Sudan (73, 74), in addition to Senegal (75), Mauritius (76) and South Africa (50, 77–79) in the Sub-Saharan region.

Additional studies on *BRCA* genes have expanded to identifying the population-based mutation frequency and screening/genetic testing in the Democratic Republic of the Congo (80), Morocco (81), Tunisia (82, 83), Algeria (84, 85), familial studies in Morocco (86, 87), and large genomic rearrangement in Egypt (88, 89). Of these, the contribution of *BRCA* mutations to male breast cancer was reported only in the Moroccan study by Guaoua et al. (86). A mutation in the *TP53* gene often accompanies *BRCA* mutations in breast and ovarian cancers, making the mutations in these DNA repair genes relevant in therapeutic interventions (90, 91). The publications on *TP53* mutation have focused on its expression in breast cancer and the contribution of its polymorphism, particularly codon 72 to breast cancer (28, 31, 33, 36, 92–94), as well as to its interaction with *MDM2* 344T>A polymorphism in response to chemotherapy of breast cancer in Tunisia (95). Other DNA repair genes that have been studied in Africa include *XRCC1* and XPD in Egypt (96, 97). Overall, even though it is one of the most studied genes in African cancer research, there remains a very small number of publications on *BRCA* mutations in the indigenous African population, clearly showing a knowledge gap on a hereditary gene critical in managing incidence and clinical outcomes in breast cancer.

Exogenous factors that drive DNA damage include viruses and xenobiotics. The presence of these agents and genetic alterations that mediate the ensuing host-response can promote carcinogenesis. The first reports of virus-associated breast cancer in Africa were by Levine et al. (98) and Hachana et al. (99), who reported the presence of a human breast carcinoma virus (a virus similar to mouse mammary tumor virus) in 74% of tumors in Tunisia. These were the only two studies that reported this virus in Africa. Studies have also shown an association of the hepatitis C virus in Egypt (100) and Human papillomavirus (HPV) in Rwanda (101) to breast cancer progression. However, the most reported virus linked to breast cancer in Africa is the Epstein-Barr virus (EBV), with studies published in Algeria (102), Eritrea (103), Egypt (104), and Sudan (105). EBV was the first identified human oncogenic virus that was detected in Uganda in 1964 by Denis Parsons Burkitt (106–108) and its molecular pathogenesis has been reviewed by Lawson et al. (109). The virus is responsible for many cancers across the continent, and the host genomic factors that facilitate tumorigenesis are described below.

The detoxification of carcinogenic chemical entities is primarily catalyzed by cytochrome P450 (phase I) and a host of phase II xenobiotic-metabolizing enzymes. Polymorphisms in these genes dictate, in large part, the effect of xenobiotics on the biological system. Such polymorphisms have been reported in *CYP1A1* and *CYP1B1* in Nigeria and Egypt (110, 111), *CYP2D6* in South Africa (112), and *CYP1A2* in Tunisia (113). Furthermore, as hormone-responsive cancers, these cytochrome P450 genes play critical roles in estrogen metabolism and the response of the tumor to endocrine therapy. For genes coding for phase II xenobiotics metabolizing enzymes, the deletion of *GSTT1* and *GSTM1* were reported by Khedhaier (114) to predict the early onset and prognosis of breast cancer among Tunisian women. The number of TA repeats in the promoter of low activity *UGT1A1* was reported to be protective against breast cancer in pre-menopausal Nigerian women (115, 116). Similarly, the association of polymorphisms in paraoxonase, cyclooxygenase, glyoxalase, and glutathione peroxidase genes with breast cancer were reported in Egypt and Rwanda (117–120).

Inflammation is a major hallmark of cancer, and it is known to contribute to aggressive tumor biology. This makes understanding the variations in immuno-oncogenic genes important in understanding the population biology of cancer in Africa. Mestiri et al. (121, 122) reported that polymorphisms in *TNF-α* and *TNFRII* increase the susceptibility to breast cancer in Tunisian women, with *TNFRII* -196R prevalent in premenopausal women. Conversely, *FASL* (rs763110) was associated with a good prognosis in the same population (123). However, *HLA-DQB1* and *HLA-G* +3142C>G (rs1063320) polymorphisms were related to increased breast cancer susceptibility (124, 125). Pathogenic polymorphisms of other inflammatory genes like *NRF2*, *IL1α*, *IL1β*, *IL6*, *IL8*, and *CXCR2* have also been identified in Tunisian and Egyptian breast cancer patients (126–130).

Recent evidence suggests that inflammation-driven cancer in Blacks is influenced by vitamin D levels (131, 132). To establish the association of vitamin D variants and related genes with breast cancer, El-Shorbagy et al. (133), Abd-Elsala et al. (134) and Shaker & Senousy (135) showed that polymorphisms in the vitamin D receptor (*VDR*) increases the risk of breast cancer in Egyptian women who carry the ATT haplotype. The risk of developing breast cancer due to these mutations was elevated in women who also carry *RANKL* (rs9533156), *OPG* (rs2073617), and *CHI3L1* (rs4950928) (135). Similar studies have also reported the risk allele in Ethiopian women as *VDR* rs2228570 (FokI) (136) but the study of Wang et al. (137) did not identify variants in vitamin D related genes as risk factors for breast cancer in Nigerian women that were used as the ancestral population for African American women. This genome-wide association study, however, identified *TYRP1* (rs41302073), a melanin synthesis regulatory gene, as a significant risk allele for breast cancer in their dataset that included African American and Barbadian women. Furthermore, the authors also used the same dataset to identify *WWCI* as an important susceptibility locus in the Hippo pathway for breast cancer (138).

Polymorphisms in the angiogenesis-associated genes have also been identified in breast cancer in African populations and include the *LEP*, *LEPR*, *VEGF*, and *MMP2*. Leptin and *LEPR* Q223R (rs1137101) were identified as risk factors for breast cancer in Egyptian and Nigerian women (139–141) while leptin alone was notably reported as a key driver of breast cancer progression through the induction of *JAK/STAT3*, *ERK1/2*, and estrogen pathways in obese Egyptian women (142). Furthermore, variants of *VEGF* and *MMPs*, which induce the upregulation of these proteins, were reported as risk factors in North African countries of Morocco, Egypt, and Tunisia (143–148). Other overexpressed angiogenic proteins reported are *EGFR* in Tunisia (149) and *IGFBP2* and *IGFBP5* in Nigerian women (150). The authors proposed these angiogenic proteins as druggable targets in breast cancer treatment. Another therapeutic pathway that has been studied is the *PIK3/AKT* pathway. Jouali et al. (151) reported *PIK3CA* hotspot mutations in 13% of triple-negative breast cancer cases in Morocco. They suggested that this pathway could be of therapeutic importance for triple-negative breast cancer in Morocco.

Cancer is a polygenic disease, and scientific investigation to understand breast cancer’s population biology, therefore, cannot be simplified to a single genetic variant. Hence, techniques to investigate multiple genes at a time such as with next generation sequencing are now being utilized to understand the genetic risk factors of breast cancer in Africa. To that effect, genome-wide studies (GWAS) published primarily on breast cancer in African populations include GWAS in Tunisia and South Africa (152–154) and whole-exome sequencing in Tunisia and Egypt (155–157). In the Tunisian population, Shan et al. (154) and Hamdi et al. (152) identified rs1219648, rs2981582, rs8051542, rs889312, and rs889312 as breast cancer susceptibility single nucleotide polymorphisms (SNPs), with rs9911630 as the SNP with the strongest effect on the expression of *BRCA1* and two long non-coding RNAs (NBR2 and LINC008854). The genome-wide copy number alteration analysis of breast cancer in South African women (153) identified the amplification in Xp22.3 and 6p21-p25, and other regions that affect known cancer genes like *CCND1*, *CDKN1A*, *MDM2*, *TP53*, and *SMAD2*. Meanwhile, the whole-exome sequencing study by Hamdi et al. (152) and Riahi et al. (156) linked breast cancer in Tunisian women to alterations in *MMS19*, *DNAH3*, *POLK*, *KATβ6*, and *RCC1* in *BRCA1/2* mutation-negative patients with familial breast cancer. A similar study in Egypt also found other novel genetic variants responsible for familial breast cancer. These genetic variants are different from those linked to DNA damage repair (like *BRCA1* and *BRCA2*) but are linked to other functional genes like *NBPF10*, *ZNF750*, *CHTI5*, *NPIPB11*, and *PHIP*, that are involved in RNA binding, transcriptional regulation, extracellular matrix, a structural protein, and signal transduction, respectively.

The contribution of epigenetic factors to risk and prognosis of breast cancer reported in Africa included the roles of tissue microRNA, circulating free mRNA, circulating long non-coding RNA (158–163) as well as DNA methylation status of breast cancer susceptibility genes like *APC*, *ERα*, *RASSFIA*, *UCHL1*, *COX-2*, and *FHIT* (161, 164–167) in breast tumor across Africa.

### Prostate Cancer

Prostate cancer continues to be the leading cause of cancer morbidity and mortality among African men (168, 169). Although genetics is a major risk factor for this disease, there are only a few publications on prostate cancer genomics in Africa. In this subsection, we review 14 papers that were relevant to prostate cancer out of the list of 375 papers extracted. Prostate cancer presents with an aggressive phenotype among men of African descent, and like breast cancer, it is a hormone-responsive tumor. Consequently, early studies on this disease identified androgen’s influence in the control of normal prostate growth and, in its transformation into adenocarcinoma, a phenomenon called the “androgen hypothesis” (170, 171). Therefore, peer-reviewed publications on prostate cancer genetics in African populations have reported genetic variants that contribute to elevated circulating androgens, including androgen reduced clearance and upregulated activity of androgen receptor. These include the polymorphisms in cytochrome P450 genes like *CYP3A4*, *CYP3A5*, *CYP1A1*, *CYP17* in Morocco, Tunisia, Nigeria, South Africa, and Senegal (172–176). Besides, alterations in CAG and GGN repeats in the androgen receptor gene have been reported as risk factors in North Africa, Ivory Coast, and Nigeria (177, 178). Unlike the North African populations, prostate cancer in Sub-Saharan African populations and North African Berbers were associated with high frequencies of low size alleles (CAG under 18 repeats, and GGC under 15 repeats) (178). Other reported genetic variations that increase African populations’ susceptibility to prostate cancer include *GSTM1*, *GSTT1*, UDP-glucuronosyltransferase, and sulfotransferase in Tunisia and Algeria (179–182).

A deeper understanding of the disease’s polygenic risk was elucidated by four studies that have investigated the genome-wide genetic variations in prostate cancer across Africa. These included GWAS of prostate cancer in Tunisia, Ghana, and Uganda (183–185), as well as a whole-genome sequencing of six individuals in South Africa (186). It is interesting to note that these four studies did not identify any common high-risk prostate cancer variants. The Tunisian study identified three regions (on chromosomes 9, 17, and 22) containing 14 significant SNPs, three of which are shared with Caucasian populations (185). The Ghanaian study of Cook et al. (184) identified 30 most significant SNPs distributed across chromosomes 1, 2, 3, 5, 6, 7, 8, 9, 10, 13, and 20.

Meanwhile, the Ugandan study identified risk alleles on chromosomes 1, 6, 11, 13, 14, and 17 (183). Although the Ugandan and Ghanaian populations shared cytoband 6p21.32 in common, the nucleotide positions and risk alleles were still different. This chromosome position codes for *HLA-DQB1*, which has been reported to be important for the adaptation of African ancestral populations to the African rainforest environment. These studies further add to the existing evidence of the heterogeneity of African populations (12) and that cancers in these populations may have a different biology. These findings provide further evidence for the need to disaggregate the Black population by genetic lineage in studying the contributions of genomics to racial disparities of diseases like prostate cancer. Importantly, it is yet to be revealed whether these differences influence the disease phenotype and disparity in outcome.

The most commonly reported genomic alteration that drives prostate tumorigenesis is *TMRPSS2-ERG* fusion, and this androgen-upregulating fusion is known to correlate with higher grades of the disease. Although men of African ancestry are known to present with higher disease grade, only three studies have examined the *TMRPSS2-ERG* fusion on the Continent (187–189). This fusion often results from either a chromosomal translocation or an interstitial deletion, and these studies reported rates that were less than 20% in Ghanaian and Black South African patients (187–189).

### Liver Cancer

According to the 2018 GLOBOCAN data, hepatocellular carcinoma accounted for 8.4 cases per 100,000 and 8.3 deaths per 100,000 globally (2) and it is the 4^th^ most common cancer in Africa. We retrieved 46 publications that studied liver cancer genetics/genomics in Africa. Several of these studies investigated the contribution of the hepatitis virus and mycotoxins to this malignancy. These biotic and abiotic agents represent the major causes of this disease on the continent (190, 191). Hence, a preponderance of publications on liver cancer in Africa focused on understanding the contribution of mutation and expression of *TP53*, and other tumor suppressors like *TP73*, *RB*, *KLF6*, and *CTNNB1*, to liver carcinogenesis (190, 192–207), particularly in Senegal, Gambia, Nigeria, South Africa, Egypt, and Morocco. These studies identified the mutation in codon 249 of *TP53* as a genetic risk factor for developing hepatocellular carcinoma following exposure to either the hepatitis virus or mycotoxins (see Lin et al. (208) for detailed mechanism). In Morocco, *MDM2* 309 T>G was associated with liver cancer (209, 210). These mutations are known to upregulate this oncogene’s expression, which in turn binds p53 and prevents its tumor suppression function (209) resulting in increased genomic instability as demonstrated by loss of heterozygosity in chromosome 4-q13 in Black South Africans (211).

The development of hepatocellular carcinoma is often preceded by chronic inflammation of the liver. In Africa, hepatic inflammation is exacerbated by high-prevent co-morbid conditions like non-alcoholic fatty liver disease (NAFLD), non-alcoholic steatohepatitis (NASH), and liver cirrhosiss. For instance, the prevalence of NAFLD in Nigeria, Ethiopia, and South Africa has been reported to be 68.8%, 73%, and 87%, respectively (212).

Despite the pervasiveness of liver cancer across Africa, only the Egyptian and Tunisian populations have been studied for the contribution of variation in inflammation-related genes to this disease. These studies reported mutations in *IL3R*, *IL17A*, *IL8*, *IL1*, *IL16*, *IL12*, *IL27*, and *TNF-α* as risk factors for hepatitis and hepatocellular carcinoma (213–219).

Other authors have focused on the development of biomarkers for liver cancer, using epigenetic factors like microRNAs. These include serum Mir-224, Mir-215, Mir-143, Mir-122, Mir-199a, and Mir-16 (220, 221). Specifically, Mir-122 and Mir-222 levels were reported by Motawi et al. (222) as a discriminating biomarker for distinguishing liver injury from liver cancer. This group further reported that LncRNA *HULC* rs7763881 and *MALAT* rs619586 were associated with decreased susceptibility of Egyptian hepatitis virus-persistent carriers to liver cancer (223).

Mycotoxicosis, with concomitant early-life protein malnutrition, is an important driver of liver cancer in Africa (224–226). One group of enzymes that are involved in detoxifying mycotoxins are the glutathione-S-transferases (μ, θ, π, α, σ). Hence, individuals who do not express all the enzymes due to homozygous deletion are more susceptible to myco-carcinogens (227). Overall, two studies have identified the deletion of *GSTM1* and *GSTT1* haplotypes as risk factors for aflatoxin-associated hepatocellular carcinoma (228, 229) in Africa.

The last group of genes that have been studied on hepatocellular carcinoma in Africa are those involved in angiogenesis, including *VEGF*, *MMP*, *RASSF1A*, and *RECK*. In Egypt, Samamoudy et al. (230) reported that patients with *MMP9* (rs3918242) are at high risk of developing liver cancer while *RECK* (rs12814325) (231) could account for the disease progression and metastasis.

### Cervical Cancer

Cervical cancer continues to be responsible for the highest cancer mortality in Africa, accounting for 2,000,000 deaths in 2018 (2), and its incidence rates continue to increase in most Sub-Saharan African countries (232). However, studies on cervical cancer genetics/genomics only represented 3% of the publications we retrieved. Similar to liver cancer, cervical cancer is viral-related and primarily caused by Human Papillomavirus (HPV). Several reviews have discussed the burden, distribution, and contribution of HPV serotypes to cervical cancer in Africa (233–235). Despite the burden of HPV in Africa, only a small proportion of women that are infected develop cervical cancer (236, 237). It is, therefore, essential to understand the genetic factors that contribute to the risk of progression from HPV infection to cervical cancer across Africa.

One of such genetic factors that increase susceptibility to HPV-associated cervical carcinogenesis is the *TP53* R72P mutation (238), which was reported in Gabon, Senegal, Sudan, Morocco, and South Africa; and this risk increases when combined with the chromosomal allelic loss of *RB* or with aberrant methylation of *DAPK1*, *RARB*, *TWIST1*, and *CDH13* (79, 239–244). Furthermore, aberrant methylation of these genes was proposed by Feng et al. (245) to be useful in Senegal for the screening of cervical cancer, either alone or in combination with cytology. The importance of this homozygous arginine polymorphism at codon 72 of *TP53* in determining genetic susceptibility of a population has been shown in Israeli Jewish women who have been reported to have reduced susceptibility to HPV-associated cervical cancer (246).

The variations in genes involved in inflammatory and apoptotic response pathways have also been reported to increase African women’s susceptibility to cervical cancer (247). The reported polymorphisms in Africa include those of *TLR 2/3/4/9* and *IL1/10/15* genes in Tunisia and -308 promoter polymorphism of *TNF-α* in South Africa (248–250). Meanwhile, polymorphisms in *FASR*-670A and *CASP8*-652 were associated with a reduced risk of developing cervical cancer in South African women (251).

### Colorectal Cancer

Colorectal cancer is the 5^th^ most common cancer in Africa and accounted for 550,000 deaths in 2018 (2). We retrieved 56 publications on colorectal cancer genetics/genomics from Nigeria, Ghana, South Africa, Algeria, Tunisia, Morocco, and Egypt. The findings in these publications included: (1) the identification of I130K *APC* polymorphism in the indigenous Black population in South Africa and Tunisia to development of familial adenomatous polyposis coli (252–255), (2) the presence of mutations in the *MUTYH*, *MLH1*, and *MSH2* gene in patients with colorectal cancer and attenuated polyposis in Algeria, Egypt, Morocco, Tunisia, and South Africa (256–266), (3) the burden of *KRAS* and *BRAF* mutations in colorectal cases in Morroco, Nigeria, Ghana, Egypt and Tunisia (267–275) and (4) the level of microsatellite instability in South African, Nigerian, Ghanian, Tunisian, and Moroccan colorectal cancer patients (259, 271, 274, 276–281). Other studies have also explored the contribution of epigenetic changes to colorectal cancer carcinogenesis in Africa (278, 282–285). For example, the methylation of *UCH1* and *p14ARF* genes were reported to drive colorectal cancer in the presence of *TP53* mutation in Tunisia (282, 283, 286, 287). Other studies on the North African populations reported the influence of polymorphisms in telomere and mitochondrial D-loop region on the clinicopathological characteristics of the colorectal cancers among their patients (288, 289). Hence, the dearth of data on the genomics of this disease makes it difficult to explain the increase in the level of sporadic colorectal cancers reported in African countries, despite the difference in lifestyle and dietary habits. Profiling of these genes, including the use of targeted next-generation sequencing, in the screening and clinical management of this disease is essential in reducing its burden (255, 290).

### Lung Cancer

Across Africa, lung cancer ranks 6^th,^ with about 550,000 cases in 2018 (2). However, the burden of this disease is on the North African countries and South Africa (2). This burden reflects the pattern of tobacco smoking reported through national surveys (291). Lung cancer genetics/genomics studies have also largely been conducted on the North African populations of Tunisia and Egypt. These studies investigated the role of angiogenic pathway genes like *EGFR* and *MMP-3* in lung carcinogenesis (292–297). The expression of *EGFR* was associated with poor prognosis, and the frequency of the mutations observed in Tunisian and Moroccan patients was similar to those of Europeans (294, 296, 298). However, Dhieh et al. (292) found that abnormal p53 expression in these patient populations was more frequent than in Europeans. Similarly, a nonsense mutation (Arg-196-Term) in exon 6 of *TP53* was identified in the small cell lung cancer from gold miners in South Africa (299).

Cigarette and air pollution are major sources of lung carcinogens; hence, studies have reported polymorphisms in *CYP1A1*, *CYP1A2*, *CYP2F1*, CYP2A6*2, and CYP2A6*9 (300–305) in lung cancer patients in North Africa. These polymorphisms alter the detoxification rate of toxicants, and individuals who carry the slow metabolizer variants have an increased risk of lung cancer (300). For example, Hussein et al. (302) concluded that Egyptian smokers with *CYP1A1* m1 (rs4646903) and *CYP1A1* m2 (rs1048943) are more likely to develop squamous cell carcinoma. Furthermore, lung carcinogens are highly inflammatory and studies in Tunisia, for example, identified alterations in inflammatory genes- *TNF-α*, *IL8*, *IL17A*, *IL17F*, *CCR2*, and VDR *Fok1* (rs2228570) and *ApaI* (rs7975232) that predispose to lung cancer (306–310).

There were additional studies that used epigenetic techniques to develop diagnostic or prognostic markers for non-small cell lung cancer in Egypt. These included the study of Haroun et al. (311) that identified *FHIT* methylation and that of Hetta et al. (312) which reported circulating microRNA-17 and microRNA-22 as potential biomarkers for early detection of lung cancer.

### Bladder Cancer

Chronic inflammation with attendant oxidative stress induced by *Schistosomia haematobium* infection remains a major cause of bladder cancer in Africa (313–315), with squamous cell carcinoma being the most common (316, 317). Schistosomiasis (or bilharzia) is a neglected tropical disease that is widespread across Africa (318). This cancer is the 10^th^ most prevalent cancer in Africa and accounted for 240,000 death in 2018. Studies on its genetics/genomics represented about 3% of the publications that we reviewed.

Its pathogenesis involves the bladder infection by *S. haemotobium*, which induces the formation of carcinogenic N-nitrosamine that contributes to squamous cell carcinogenesis (319), particularly in individuals with *TP53* mutation (320). In addition, mutations in genes associated with inflammation and detoxification of carcinogenesis are critical risk factors. One of which is the polymorphisms in *CYP2D6* and *CYP1A1* that have been studied in Egypt and Tunisia (321–323) and that of CYP2D*1A, which was found to increase the risk and clinicopathological outcome of both transitional and squamous cell carcinomas in Egypt (322). Similar findings were reported in the same North African countries for individuals with *GST* null genotypes and NAT*5 (341T>C) (324–331).

The neoplastic transformation and progression of bladder cancer are enhanced through oxidative stress-induced genomic instability and chromosomal aberrations, which particularly involve the loss of heterozygosity on chromosomes 8 and 9 (332–338). These aberrations, coupled with p53 and p16 loss, have been reported in both bilharzial and non-bilharzial bladder cancer in Egypt and Tunisia (36, 332, 339–343).

The pattern of CpG island hypermethylation was studied by Gustierrez et al. (344) and they showed that the Schistosoma-associated tumors in Egyptian patients had higher hypermethylation of genes like E-cadherin, DAP-kinase, *TP14*, *TP15*, *TP16*, *APC*, *GSTP1*, and *TP73*. Other authors have further proposed using these unique epigenetic modifications for the early diagnosis of bladder cancer by utilizing plasma circulating microRNA and urinary DNA methylation profile (345, 346).

It is important to note that pesticides have also been implicated in bladder tumorigenesis (347, 348) through oxidative stress and *KRAS* mutation in Egyptian occupationally-exposed individuals (347).

### Other Solid Tumors

Studies in South Africa, Egypt, Sudan, and Tunisia identified the EBV as the major cause of head and neck cancer (349–354). The genetic risk factors that have been reported include *TP53* mutations in Sudan and Egypt (355–357), *XRCC1*, *TNF-α*, *IL10* promoter, *CYP1A1*, *CYP2D6*, and *NAT2* polymorphisms in Tunisia (358–361) as well as genome-wide aberrations associated with chromosomes 2p, 3p, 5q, and 18q and microsatelite instabilities (362–364) and mutations in the mitochondrial D-Loop region and Cytochrome b gene (365).

The genomic studies on the cancer of the brain, kidney, pancreas, and other organs are still emerging with very limited publications (366–378). The emphasis of these publications on the polymorphisms of genes associated with inflammatory response is an indication of the importance of this biological process to the neoplastic transformation of normal tissue and the progression of the malignancy. In addition, studies on retinoblastoma concentrated on identifying the constitutional mutations in *RB* within the North African populations (379–382) while publications on esophageal and gastric cancers focused on identifying the role of *RAS* genes mutations as drivers of genomic instability (383–386).

### Lymph and Hematological Malignancies

The most prevalent lymphoma in Africa is Burkitt lymphoma. Its pattern and geographical spread are similar to that of malaria and ancient human migration on the continent (387–392). This aggressive pediatric B-cell non-Hodgkin lymphoma is caused by the EBV, which induces genomic instability in the B-cell that results in hyperproliferation (393, 394) and it is associated with unique *TP53* mutations that are clustered between codons 213 to 248 (395–397).

Other studies on lymphoma include: (1) the role of *TP73* and *FOXP3* in the pathogenesis of reactive lymphoid hyperplasia and diffuse B-cell lymphoma, as well as the contribution of *HLA-G* polymorphism to non-Hodgkin lymphoma in Egypt (398–400), (2) susceptibility of individuals with A/A genotype of *TNF* promoter (-308A/G) to non-Hodgkins lymphoma in Tunisia (401) and Egypt (402) and the identification of HLA-B*18, DRB1*03, DRB1*07, and DQB1*02 as lymphoma susceptibility loci in Algerian children (403).

Studies from Egypt, Tunisia, and Morocco have identified the susceptibility or prognostic implications of mutations in *FLT3-ITD*, *NPM-1*, *KIT*, *NPM1*, *HFE*, *DNMT3A*, *TERT*, and *NRAS* in hematological malignancies (404–410). *NRAS* G12D and *NRAS* G13C mutations were reported in Nigerian leukemia patients Anyanwu et al. (411).

## Discussion

In order to provide an overview of research progress in African cancer genomics with the view of identifying the critical gaps, we searched and reviewed publications on cancer genetics and genomics in Africa. The 375 publications on cancer genetics/genomics retrieved on PubMed represented only 0.016% of total publications on cancer globally.

According to the 2018 GLOBOCAN data on cancer in Africa, the most frequently diagnosed cancers were breast, cervix, prostate, liver, and colorectum, while the leading causes of cancer deaths were from cancers of the cervix, breast, prostate, liver, and colorectum (2). However, of the top ten frequently diagnosed cancers and the leading cause of cancer deaths in Africa, only breast, colorectal, liver, and ovarian cancers were proportionately represented in cancer genetics/genomics studies returned from search terms.

Overall, Africans are grossly underrepresented in cancer genomics and molecular biology research globally. For example, research on prostate cancer in African men or breast cancer in African women, both leading causes of death in Africa, are still understudied compared to cancers in their non-Black and white counterparts (412).

Although Africa seems to be on the right track in terms of focusing on some of the top cancers, researchers and funding agencies, need to elevate and prioritize genetics and genomics research on cancers that remain hugely underrepresented or unrepresented in the literature for which there is a significant burden in Africa. These include cancers of the lung, ovary, stomach, bladder, prostate, and non-Hodgkin lymphoma, which are among the leading ten causes of death but remain understudied in the literature. Filling this research gap is essential to improving awareness, prevention, diagnosis, and treatment outcomes for people affected by cancer across the continent.

It is also worth noting that most studies on cancer in Africa are clustered to a few regions, mainly North Africa, Nigeria, Ghana, and South Africa. Most of the continent lacks any appreciable data, is often excluded from research efforts, and is devoid of the infrastructure and resources needed to contribute to cancer genomics/genetics discoveries.

It is important to reiterate that this review was based on publications that were indexed in Pubmed only. This is because Pubmed is considered as the most reputable index for biomedical publications, and the data we have retrieved are a good representation of the spectrum and scope of this review. It is also possible that our search did not retrieve some studies that included African populations, and this could be because those studies were not focused on African countries or groups but have used them for comparative purposes, thereby making the data obscure and less prominent in their findings. The use of MeSH terms ensured that relevant publications were extracted from Pubmed.

## Conclusion and Future Directions

As presented in this review, the preponderance of the peer-reviewed publications on cancer genomics in Africa was on the North Africa populations. Hence, there is a need for a concerted effort to address the gaps in the contribution of genomic variance and alterations to cancer in Sub-Saharan African populations. Recently, Durvasula and Sankararaman (413) reported the presence of ghost archaic introgression into the genome of Sub-Saharan Africa populations, and some of this introgression included regions involved in carcinogenesis. This and the details presented in this review lay credence to the inadequacy of the use of predominantly Caucasian genomics data for cancer control in Africa. The use of personalized medicine and targeted therapy in cancer management rely on understanding the genomics of the population. Hence, there is a need to step up cancer genomics studies for Africa to benefit from medical advances. Also, because Africa is the root of humanity, understanding the genetic basis of this disease in Africans will contribute to improving cancer health equity globally.

In addition, scientific investigations on cancer racial disparity have largely considered the Black race as a homogenous group. However, the evidence is now emerging that there are within-group differences in cancer risk among Blacks (414). This review also clearly demonstrated the need to disaggregate Africa in cancer studies. To reduce cancer disparity and achieve equity in treatment outcomes, cancer genetics and genomics studies in African should endeavor to stratify populations by their ancestry roots, tribes, or languages rather than countries. This is imperative to identifying population-relevant genetic variants since African countries are geopolitical constructs that bear no relationship with the biological relatedness of the people that are clustered together in those countries.

Furthermore, every genomic study requires a reference to make an appropriate inference, but African populations are presently inadequately represented in the current reference genomes. To address this unmet need, Shermanet et al. (415) recently published a pan-African reference genome. The African Pan Genome sequences they assembled revealed that up to 10% of the genome will be missed by any efforts relying only on GRCh38 to study human variation. Yet, it is important to note that their study only included representative samples (5%) from Ibadan, Nigeria, and may not be a true “Pan African Genome” and may best represent the West African human population, which the Yoruba people belong to. Further research efforts are, therefore, needed to assemble more African reference genomes, which should be based on the genetic divergence of human populations in Africa.

## Author Contributions

BS conceived, designed, and supervised the review. SR and OR collected and analyzed the data. BS, SR, and OR wrote the manuscript. All authors contributed to the article and approved the submitted version.

## Funding

Fulbright Visiting Scholar Fellowship (Grant ID: E0604356) awarded to SR.

## Conflict of Interest

The authors declare that the research was conducted in the absence of any commercial or financial relationships that could be construed as a potential conflict of interest.

## References

[1] Organization WH. Global Health Observatory: Non-communicable diseases mortality and morbidity. (2013). Available at: https://www.who.int/docs/default-source/gho-documents/world-health-statistic-reports/who-his-hsi-13-1-eng.pdf.

[2] Bray F Ferlay J Soerjomataram I Siegel RL Torre LA Jemal A. Global cancer statistics 2018: GLOBOCAN estimates of incidence and mortality worldwide for 36 cancers in 185 countries. CA Cancer J Clin (2018) 68(6):394–424. 10.3322/caac.21492 30207593

[3] Walsh R Goh B-C. Population diversity in oncology drug responses and implications to drug development. Chin Clin Oncol (2019) 8(3):3. 10.21037/cco.2019.05.01 31311278

[4] Teh BT. The importance of including diverse populations in cancer genomic and epigenomic studies. Nat Rev Cancer (2019) 19(7):361–2. 10.1038/s41568-019-0158-0 31160734

[5] Haiman CA Stram DO. Exploring genetic susceptibility to cancer in diverse populations. Curr Opin Genet Dev (2010) 20(3):330–5. 10.1016/j.gde.2010.02.007 PMC4196678 20359883

[6] Tishkoff SA Reed FA Friedlaender FR Ehret C Ranciaro A Froment A. The genetic structure and history of Africans and African Americans. Science (2009) 324(5930):1035–44. 10.1126/science.1172257 PMC2947357 19407144

[7] Lachance J Vernot B Elbers CC Ferwerda B Froment A Bodo JM. Evolutionary history and adaptation from high-coverage whole-genome sequences of diverse African hunter-gatherers. Cell (2012) 150(3):457–69. 10.1016/j.cell.2012.07.009 PMC3426505 22840920

[8] Campbell MC Hirbo JB Townsend JP Tishkoff SA. The peopling of the African continent and the diaspora into the new world. Curr Opin Genet Dev (2014) 29:120–32. 10.1016/j.gde.2014.09.003 PMC4308437 25461616

[9] Tucci S Akey JM. The long walk to African genomics. Genome Biol (2019) 20(1):130. 10.1186/s13059-019-1740-1 31248437 PMC6598360

[10] Yuan J Hu Z Mahal BA Zhao SD Kensler KH Pi J. Integrated analysis of genetic ancestry and genomic alterations across cancers. Cancer Cell (2018) 34(4):549–60.e9. 10.1016/j.ccell.2018.08.019 30300578 PMC6348897

[11] Quach H Quintana-Murci L. Living in an adaptive world: Genomic dissection of the genus Homo and its immune response. J Exp Med (2017) 214(4):877–94. 10.1084/jem.20161942 PMC5379985 28351985

[12] Schlebusch CM Malmstrom H Gunther T Sjodin P Coutinho A Edlund H. Southern African ancient genomes estimate modern human divergence to 350,000 to 260,000 years ago. Science (2017) 358(6363):652–5. 10.1126/science.aao6266 28971970

[13] Gurdasani D Carstensen T Tekola-Ayele F Pagani L Tachmazidou I Hatzikotoulas K. The African Genome Variation Project shapes medical genetics in Africa. Nature (2015) 517(7534):327–32. 10.1038/nature13997 PMC4297536 25470054

[14] Gurdasani D Barroso I Zeggini E Sandhu MS. Genomics of disease risk in globally diverse populations. Nat Rev Genet (2019) 20(9):520–35. 10.1038/s41576-019-0144-0 31235872

[15] Peprah E Wiley K Sampson U Narula J. A new age for african-driven genomics research: human heredity and health in Africa (H3Africa). Glob Heart (2017) 12(2):67–8. 10.1016/j.gheart.2017.05.003 28867289

[16] Mao Y Lu Z. MeSH Now: automatic MeSH indexing at PubMed scale via learning to rank. J BioMed Semantics (2017) 8(1):15. 10.1186/s13326-017-0123-3 28412964 PMC5392968

[17] Kovalchik S. RISmed: download content from NCBI databases. R package version, Vol. 2. (2015).

[18] Rani J Ramachandran S. pubmed. mineR: An R package with text-mining algorithms to analyse PubMed abstracts. J Biosci (2015) 40(4):671–82. 10.1007/s12038-015-9552-2 26564970

[19] Acharya A Li S Liu X Pelekos G Ziebolz D Mattheos N. Biological links in periodontitis and rheumatoid arthritis: Discovery via text-mining PubMed abstracts. J Periodontal Res (2019) 54(4):318–28. 10.1111/jre.12632 30536918

[20] Povey S Lovering R Bruford E Wright M Lush M Wain H. The HUGO Gene Nomenclature Committee (HGNC). Hum Genet (2001) 109(6):678–80. 10.1007/s00439-001-0615-0 11810281

[21] Salicru M Ocana J Sanchez-Pla A. Comparison of lists of genes based on functional profiles. BMC Bioinf (2011) 12:401. 10.1186/1471-2105-12-401 PMC3747174 21999355

[22] Gamberoni G Storari S Volinia S. Finding biological process modifications in cancer tissues by mining gene expression correlations. BMC Bioinf (2006) 7:6. 10.1186/1471-2105-7-6 PMC1360676 16401337

[23] Faguet GB. A brief history of cancer: age-old milestones underlying our current knowledge database. Int J Cancer (2015) 136(9):2022–36. 10.1002/ijc.29134 25113657

[24] Ades F Tryfonidis K Zardavas D. The past and future of breast cancer treatment-from the papyrus to individualised treatment approaches. Ecancermedicalscience (2017) 11:746. 10.3332/ecancer.2017.746 28690677 PMC5481194

[25] Paul A Paul S. The breast cancer susceptibility genes (BRCA) in breast and ovarian cancers. Front Biosci (Landmark Ed) (2014) 19:605–18. 10.2741/4230 PMC4307936 24389207

[26] Kreiss Y Barak F Baruch RG Levy-Lahad E Pras E Friedman E. The founder mutations in the BRCA1, BRCA2, and ATM genes in Moroccan Jewish women with breast cancer. Genet Test (2000) 4(4):403–7. 10.1089/109065700750065171 11216667

[27] Mestiri S Monastiri K Ben Ahmed S Bouaouina N Presneau N Bignon YJ. [Mutational analysis of breast/ovarian cancer hereditary predisposition gene BRCA1 in Tunisian women]. Arch Inst Pasteur Tunis (2000) 77(1-4):11–5. 14658222

[28] Masri MA Abdel Seed NM Fahal AH Romano M Baralle F El Hassam AM. Minor role for BRCA2 (exon11) and p53 (exon 5-9) among Sudanese breast cancer patients. Breast Cancer Res Treat (2002) 71(2):145–7. 10.1023/a:1013807830329 11883440

[29] el AHT Khalifa A Kamel AS. Immunohistochemical expression of p53 and c-erbB2 proteins in breast cancer in Egypt. Anticancer Res (2000) 20(3B):2145–50. 10928168

[30] Monastiri K Ben Ahmed S Presneau N Bignon JY Chouchane L. [Rapid detection of BRCA-1 germline mutations by the protein truncation test in Tunisian families]. Tunis Med (2002) 80(9):515–8. 12632763

[31] Hussein MR Ismael HH. Alterations of p53, Bcl-2, and hMSH2 protein expression in the normal breast, benign proliferative breast disease, in situ and infiltrating ductal breast carcinomas in the upper Egypt. Cancer Biol Ther (2004) 3(10):983–8. 10.4161/cbt.3.10.1136 15467428

[32] Swellam M Ismail M Eissa S Hamdy M Mokhtar N. Emerging role of p53, bcl-2 and telomerase activity in Egyptian breast cancer patients. IUBMB Life (2004) 56(8):483–90. 10.1080/15216540400010834 15545228

[33] Saleh EM Wahab AH Elhouseini ME Eisa SS. Loss of heterozygosity at BRCA1, TP53, nm-23 and other loci on chromosome 17q in human breast carcinoma. J Egypt Natl Canc Inst (2004) 16(1):62–8. 15717000

[34] Troudi W Uhrhammer N Sibille C Dahan C Mahfoudh W Bouchlaka Souissi C. Contribution of the BRCA1 and BRCA2 mutations to breast cancer in Tunisia. J Hum Genet (2007) 52(11):915–20. 10.1007/s10038-007-0195-5 17922257

[35] Troudi W Uhrhammer N Ben Romdhane K Sibille C Mahfoudh W Chouchane L. Immunolocalization of BRCA1 protein in tumor breast tissue: prescreening of BRCA1 mutation in Tunisian patients with hereditary breast cancer? Eur J Histochem (2007) 51(3):219–26. 17921118

[36] Mabrouk I Baccouche S El-Abed R Mokdad-Gargouri R Mosbah A Said S. No evidence of correlation between p53 codon 72 polymorphism and risk of bladder or breast carcinoma in Tunisian patients. Ann N Y Acad Sci (2003) 1010:764–70. 10.1196/annals.1299.137 15033824

[37] Charef-Hamza S Trimeche M Ziadi S Amara K Gaddas N Mokni M. Loss of heterozygosity at the BRCA1 locus in Tunisian women with sporadic breast cancer. Cancer Lett (2005) 224(2):185–91. 10.1016/j.canlet.2004.11.001 15914269

[38] Karray-Chouayekh S Trifa F Khabir A Boujelbane N Sellami-Boudawara T Daoud J. Clinical significance of epigenetic inactivation of hMLH1 and BRCA1 in Tunisian patients with invasive breast carcinoma. J BioMed Biotechnol (2009) 2009:369129. 10.1155/2009/369129 19644562 PMC2717605

[39] Uhrhammer N Abdelouahab A Lafarge L Feillel V Ben Dib A Bignon YJ. BRCA1 mutations in Algerian breast cancer patients: high frequency in young, sporadic cases. Int J Med Sci (2008) 5(4):197–202. 10.7150/ijms.5.197 18645608 PMC2452980

[40] Awadelkarim KD Aceto G Veschi S Elhaj A Morgano A Mohamedani AA. BRCA1 and BRCA2 status in a Central Sudanese series of breast cancer patients: interactions with genetic, ethnic and reproductive factors. Breast Cancer Res Treat (2007) 102(2):189–99. 10.1007/s10549-006-9303-z 17333343

[41] McCann KE Hurvitz SA. Advances in the use of PARP inhibitor therapy for breast cancer. Drugs Context (2018) 7:212540. 10.7573/dic.212540 30116283 PMC6089618

[42] Heramb C Wangensteen T Grindedal EM Ariansen SL Lothe S Heimdal KR. BRCA1 and BRCA2 mutation spectrum - an update on mutation distribution in a large cancer genetics clinic in Norway. Hered Cancer Clin Pract (2018) 16:3. 10.1186/s13053-017-0085-6 29339979 PMC5761139

[43] Silverman TB Kuperman GJ Vanegas A Sin M Dimond J Crew KD. An applied framework in support of shared decision making about BRCA genetic testing. AMIA Annul Symp Proc (2018) 961–9. PMC6371283 30815139

[44] Rebbeck TR Friebel TM Friedman E Hamann U Huo D Kwong A. Mutational spectrum in a worldwide study of 29,700 families with BRCA1 or BRCA2 mutations. Hum Mutat (2018) 39(5):593–620. 10.1002/humu.23406 29446198 PMC5903938

[45] Zhang J Fackenthal JD Huo D Zheng Y Olopade OI. Searching for large genomic rearrangements of the BRCA1 gene in a Nigerian population. Breast Cancer Res Treat (2010) 124(2):573–7. 10.1007/s10549-010-1006-9 20596889

[46] Zhang J Fackenthal JD Zheng Y Huo D Hou N Niu Q. Recurrent BRCA1 and BRCA2 mutations in breast cancer patients of African ancestry. Breast Cancer Res Treat (2012) 134(2):889–94. 10.1007/s10549-012-2136-z 22739995

[47] Zheng Y Walsh T Gulsuner S Casadei S Lee MK Ogundiran TO. Inherited breast cancer in Nigerian women. J Clin Oncol (2018) 36(28):2820–5. 10.1200/JCO.2018.78.3977 PMC6161833 30130155

[48] Fackenthal JD Zhang J Zhang B Zheng Y Hagos F Burrill DR. High prevalence of BRCA1 and BRCA2 mutations in unselected Nigerian breast cancer patients. Int J Cancer (2012) 131(5):1114–23. 10.1002/ijc.27326 22034289

[49] Pitt JJ Riester M Zheng Y Yoshimatsu TF Sanni A Oluwasola O. Characterization of Nigerian breast cancer reveals prevalent homologous recombination deficiency and aggressive molecular features. Nat Commun (2018) 9(1):4181. 10.1038/s41467-018-06616-0 30327465 PMC6191428

[50] Pegoraro RJ Moodley M Rom L Chetty R Moodley J. P53 codon 72 polymorphism and BRCA 1 and 2 mutations in ovarian epithelial malignancies in black South Africans. Int J Gynecol Cancer (2003) 13(4):444–9. 10.1046/j.1525-1438.2003.13333.x 12911720

[51] Mahfoudh W Bettaieb I Ghedira R Snoussi K Bouzid N Klayech Z. Contribution of BRCA1 5382insC mutation in triple negative breast cancer in Tunisia. J Transl Med (2019) 17(1):123. 10.1186/s12967-019-1873-8 30975216 PMC6458830

[52] Reeves MD Yawitch TM van der Merwe NC van den Berg HJ Dreyer G van Rensburg EJ. BRCA1 mutations in South African breast and/or ovarian cancer families: evidence of a novel founder mutation in Afrikaner families. Int J Cancer (2004) 110(5):677–82. 10.1002/ijc.20186 15146556

[53] van der Merwe NC Hamel N Schneider SR Apffelstaedt JP Wijnen JT Foulkes WD. A founder BRCA2 mutation in non-Afrikaner breast cancer patients of the Western Cape of South Africa. Clin Genet (2012) 81(2):179–84. 10.1111/j.1399-0004.2010.01617.x 21204799

[54] Sluiter M Mew S van Rensburg EJ. PALB2 sequence variants in young South African breast cancer patients. Fam Cancer (2009) 8(4):347–53. 10.1007/s10689-009-9241-0 19333784

[55] Zhang B Fackenthal JD Niu Q Huo D Sveen WE DeMarco T. Evidence for an ancient BRCA1 mutation in breast cancer patients of Yoruban ancestry. Fam Cancer (2009) 8(1):15–22. 10.1007/s10689-008-9205-9 18679828

[56] Diez O Pelegri A Gadea N Gutierrez-Enriquez S Masas M Tenes A. Novel BRCA1 deleterious mutation (c.1949_1950delTA) in a woman of Senegalese descent with triple-negative early-onset breast cancer. Oncol Lett (2011) 2(6):1287–9. 10.3892/ol.2011.390 PMC3406549 22848303

[57] Cherbal F Bakour R Adane S Boualga K. BRCA1 and BRCA2 germline mutation spectrum in hereditary breast/ovarian cancer families from Maghrebian countries. Breast Dis (2012) 34(1):1–8. 10.3233/bd-130348 23697973

[58] Abdel-Mohsen MA Ahmed OA El-Kerm YM. BRCA1 gene mutations and influence of chemotherapy on autophagy and apoptotic mechanisms in Egyptian breast cancer patients. Asian Pac J Cancer Prev (2016) 17(3):1285–92. 10.7314/apjcp.2016.17.3.1285 27039761

[59] Riahi A Gourabi ME Chabouni-Bouhamed H. Dissimilarity between sporadic, non-BRCA1/2 families and hereditary breast cancer, linked to BRCA genes, in the Tunisian population. Breast Cancer (2016) 23(5):807–12. 10.1007/s12282-015-0648-1 26476744

[60] Hadiji-Abbes N Trifa F Choura M Khabir A Sellami-Boudawara T Frikha M. A novel BRCA2 in frame deletion in a Tunisian woman with early onset sporadic breast cancer. Pathol Biol (Paris) (2015) 63(4-5):185–9. 10.1016/j.patbio.2015.07.009 26320393

[61] Troudi W Loueslati B Baccar A Ben Ayed F Ben Ammar El Gaaied A. [Penetrance of BRCA1 gene mutation and DNA mitochondrial in Tunisian breast cancer occurrence]. Tunis Med (2009) 87(8):494–8. 20180350

[62] Troudi W Uhrhammer N Romdhane KB Sibille C Amor MB Khodjet El Khil H. Complete mutation screening and haplotype characterization of BRCA1 gene in Tunisian patients with familial breast cancer. Cancer Biomark (2008) 4(1):11–8. 10.3233/cbm-2008-4102 18334730

[63] Riahi A Ghourabi ME Fourati A Chaabouni-Bouhamed H. Family history predictors of BRCA1/BRCA2 mutation status among Tunisian breast/ovarian cancer families. Breast Cancer (2017) 24(2):238–44. 10.1007/s12282-016-0693-4 27025497

[64] Riahi A Kharrat M Ghourabi ME Khomsi F Gamoudi A Lariani I. Mutation spectrum and prevalence of BRCA1 and BRCA2 genes in patients with familial and early-onset breast/ovarian cancer from Tunisia. Clin Genet (2015) 87(2):155–60. 10.1111/cge.12337 24372583

[65] Riahi A Kharrat M Lariani I Chaabouni-Bouhamed H. High-resolution melting (HRM) assay for the detection of recurrent BRCA1/BRCA2 germline mutations in Tunisian breast/ovarian cancer families. Fam Cancer (2014) 13(4):603–9. 10.1007/s10689-014-9740-5 25069718

[66] Mahfoudh W Bouaouina N Ahmed SB Gabbouj S Shan J Mathew R. Hereditary breast cancer in Middle Eastern and North African (MENA) populations: identification of novel, recurrent and founder BRCA1 mutations in the Tunisian population. Mol Biol Rep (2012) 39(2):1037–46. 10.1007/s11033-011-0829-8 PMC3249560 21603858

[67] Henouda S Bensalem A Reggad R Serrar N Rouabah L Pujol P. Contribution of BRCA1 and BRCA2 germline mutations to early Algerian breast cancer. Dis Markers (2016) 2016:7869095. 10.1155/2016/7869095 26997744 PMC4779828

[68] Cherbal F Bakour R Adane S Boualga K Benais-Pont G Maillet P. BRCA1 and BRCA2 germline mutations screening in Algerian breast/ovarian cancer families. Dis Markers (2010) 28(6):377–84. 10.3233/dma-2010-0718 PMC3833328 20683152

[69] Cherbal F Salhi N Bakour R Adane S Boualga K Maillet P. BRCA1 and BRCA2 unclassified variants and missense polymorphisms in Algerian breast/ovarian cancer families. Dis Markers (2012) 32(6):343–53. 10.3233/dma-2012-0893 PMC3826381 22684231

[70] Quiles F Teule A Martinussen Tandstad N Feliubadalo L Tornero E Del Valle J. Identification of a founder BRCA1 mutation in the Moroccan population. Clin Genet (2016) 90(4):361–5. 10.1111/cge.12747 26864382

[71] Jouhadi H Tazzite A Azeddoug H Naim A Nadifi S Benider A. Clinical and pathological features of BRCA1/2 tumors in a sample of high-risk Moroccan breast cancer patients. BMC Res Notes (2016) 9:248. 10.1186/s13104-016-2057-8 27129401 PMC4850715

[72] Laarabi FZ Ratbi I Elalaoui SC Mezzouar L Doubaj Y Bouguenouch L. High frequency of the recurrent c.1310_1313delAAGA BRCA2 mutation in the North-East of Morocco and implication for hereditary breast-ovarian cancer prevention and control. BMC Res Notes (2017) 10(1):188. 10.1186/s13104-017-2511-2 28577564 PMC5457611

[73] Biunno I Aceto G Awadelkarim KD Morgano A Elhaj A Eltayeb EA. BRCA1 point mutations in premenopausal breast cancer patients from Central Sudan. Fam Cancer (2014) 13(3):437–44. 10.1007/s10689-014-9717-4 24729269

[74] Elimam AA Aabdein M Eldeen MEM Altayb HN Taha MA Nimir MN. Monoallelic characteristic-bearing heterozygous L1053X in BRCA2 gene among Sudanese women with breast cancer. BMC Med Genet (2017) 18(1):85. 10.1186/s12881-017-0448-x 28814288 PMC5559773

[75] Diop JPD Diallo RN Bourdon-Huguenin V Dem A Diouf D Dieng MM. Novel BRCA2 pathogenic variant c.5219 T > G; p.(Leu1740Ter) in a consanguineous Senegalese family with hereditary breast cancer. BMC Med Genet (2019) 20(1):73. 10.1186/s12881-019-0814-y 31060517 PMC6501405

[76] Khittoo G Manning A Mustun H Appadoo J Venkatasamy S Fagoonee I. Mutation analysis of a Mauritian hereditary breast cancer family reveals the BRCA2 6503deITT mutation previously found to recur in different ethnic populations. Hum Hered (2001) 52(1):55–8. 10.1159/000053354 11359068

[77] Francies FZ Wainstein T De Leeneer K Cairns A Murdoch M Nietz S. BRCA1, BRCA2 and PALB2 mutations and CHEK2 c.1100delC in different South African ethnic groups diagnosed with premenopausal and/or triple negative breast cancer. BMC Cancer (2015) 15:912. 10.1186/s12885-015-1913-6 26577449 PMC4647511

[78] Yawitch TM van Rensburg EJ Mertz M Falkson CI. Absence of commonly recurring BRCA1 mutations in black South African women with breast cancer. S Afr Med J (2000) 90(8):788. 11022626

[79] Pegoraro RJ Rom L Lanning PA Moodley M Naiker S Moodley J. P53 codon 72 polymorphism and human papillomavirus type in relation to cervical cancer in South African women. Int J Gynecol Cancer (2002) 12(4):383–8. 10.1046/j.1525-1438.2002.01109.x 12144687

[80] Luyeye Mvila G Postema S Marchal G Van Limbergen E Verdonck F Matthijs G. From the set-up of a screening program of breast cancer patients to the identification of the first BRCA mutation in the DR Congo. BMC Public Health (2014) 14:759. 10.1186/1471-2458-14-759 25070656 PMC4133620

[81] El Khachibi M Diakite B Hamzi K Badou A Senhaji MA Bakhchane A. Screening of exon 11 of BRCA1 gene using the high resolution melting approach for diagnosis in Moroccan breast cancer patients. BMC Cancer (2015) 15:81. 10.1186/s12885-015-1040-4 25885115 PMC4351675

[82] Riahi A Chabouni-Bouhamed H Kharrat M. Prevalence of BRCA1 and BRCA2 large genomic rearrangements in Tunisian high risk breast/ovarian cancer families: Implications for genetic testing. Cancer Genet (2017) 210:22–7. 10.1016/j.cancergen.2016.11.002 28212807

[83] Fourati A Louchez MM Fournier J Gamoudi A Rahal K El May MV. Screening for common mutations in BRCA1 and BRCA2 genes: interest in genetic testing of Tunisian families with breast and/or ovarian cancer. Bull Cancer (2014) 101(11):E36–40. 10.1684/bdc.2014.2049 25418591

[84] Mehemmai C Cherbal F Hamdi Y Guedioura A Benbrahim W Bakour R. BRCA1 and BRCA2 germline mutation analysis in hereditary breast/ovarian cancer families from the aures region (Eastern Algeria): first report. Pathol Oncol Res (2019) 26(2):715–26. 10.1007/s12253-019-00586-4 30715675

[85] Boulenouar ACS Coulet F Bendiab FMT Boudinar FZ Senhadji R. BRCA1 and BRCA2 germline mutation screening in Western Algeria using high resolution melting analysis (HRM). Gulf J Oncolog (2018) 1(27):31–7. 30145549

[86] Guaoua S Ratbi I Lyahyai J El Alaoui SC Laarabi FZ Sefiani A. Novel nonsense mutation of BRCA2 gene in a Moroccan man with familial breast cancer. Afr Health Sci (2014) 14(2):468–71. 10.4314/ahs.v14i2.25 PMC4196389 25320599

[87] Laraqui A Uhrhammer N Lahlou-Amine I El Rhaffouli H El Baghdadi J Dehayni M. Mutation screening of the BRCA1 gene in early onset and familial breast/ovarian cancer in Moroccan population. Int J Med Sci (2013) 10(1):60–7. 10.7150/ijms.5014 PMC3534878 23289006

[88] Eid OM El Ghoroury EA Eid MM Mahrous RM Abdelhamid MI Aboafya ZI. Evaluation of BRCA1 large genomic rearrangements in group of Egyptian female breast cancer patients using MLPA. Gulf J Oncolog (2017) 1(25):64–9. 29019333

[89] Hagag E Shwaireb M Coffa J El Wakil A. Screening for BRCA1 large genomic rearrangements in female Egyptian hereditary breast cancer patients. East Mediterr Health J (2013) 19(3):255–62. 10.26719/2013.19.3.255 23879077

[90] Na B Yu X Withers T Gilleran J Yao M Foo TK. Therapeutic targeting of BRCA1 and TP53 mutant breast cancer through mutant p53 reactivation. NPJ Breast Cancer (2019) 5:14. 10.1038/s41523-019-0110-1 30993195 PMC6465291

[91] Rose SL Buller RE. The role of p53 mutation in BRCA1-associated ovarian cancer. Minerva Ginecol (2002) 54(3):201–9. 12063435

[92] Aceto GM Awadelkarim KD Di Nicola M Moscatello C Pantalone MR Verginelli F. Germline TP53 mutation spectrum in Sudanese premenopausal breast cancer patients: correlations with reproductive factors. Breast Cancer Res Treat (2019) 175(2):479–85. 10.1007/s10549-019-05168-1 PMC6533225 30796655

[93] El-Ghannam DM Arafa M Badrawy T. Mutations of p53 gene in breast cancer in the Egyptian province of Dakahliya. J Oncol Pharm Pract (2011) 17(2):119–24. 10.1177/1078155209356130 20015931

[94] Habyarimana T Attaleb M Mugenzi P Mazarati JB Bakri Y El Mzibri M. Association of p53 codon 72 polymorphism with breast cancer in a rwandese population. Pathobiology (2018) 85(3):186–91. 10.1159/000481664 29131100

[95] Arfaoui A Douik H Kablouti G Chaaben A Handiri N Zid Z. MDM2 344T>A polymorphism; could it be a predictive marker of anthracycline resistance? J BUON Off J Balkan Union Oncol (2016) 21(3):732–9. 27569097

[96] Ramadan RA Desouky LM Elnaggar MA Moaaz M Elsherif AM. Association of DNA repair genes XRCC1 (Arg399Gln), (Arg194Trp) and XRCC3 (Thr241Met) polymorphisms with the risk of breast cancer: a case-control study in Egypt. Genet testing Mol Biomarkers (2014) 18(11):754–60. 10.1089/gtmb.2014.0191 25340946

[97] Hussien YM Gharib AF Awad HA Karam RA Elsawy WH. Impact of DNA repair genes polymorphism (XPD and XRCC1) on the risk of breast cancer in Egyptian female patients. Mol Biol Rep (2012) 39(2):1895–901. 10.1007/s11033-011-0935-7 21643959

[98] Levine PH Pogo BG Klouj A Coronel S Woodson K Melana SM. Increasing evidence for a human breast carcinoma virus with geographic differences. Cancer (2004) 101(4):721–6. 10.1002/cncr.20436 15305401

[99] Hachana M Trimeche M Ziadi S Amara K Gaddas N Mokni M. Prevalence and characteristics of the MMTV-like associated breast carcinomas in Tunisia. Cancer Lett (2008) 271(2):222–30. 10.1016/j.canlet.2008.06.001 18639977

[100] Attallah AM El-Far M Abdelrazek MA Omran MM Mahmoud AZ Khalifa HS. HCV nonstructural protein 4 is associated with aggressiveness features of breast cancer. Breast Cancer (2018) 25(3):297–302. 10.1007/s12282-017-0829-1 29285674

[101] Habyarimana T Attaleb M Mazarati JB Bakri Y El Mzibri M. Detection of human papillomavirus DNA in tumors from Rwandese breast cancer patients. Breast Cancer (2018) 25(2):127–33. 10.1007/s12282-018-0831-2 29350329

[102] Yahia R Zaoui C Derbale W Boudi H Chebloune Y Sahraoui T. Epstein Barr virus and invasive mammary carcinomas: EBNA, EBERs and molecular profile in a population of West Algeria. Annales biologie clinique (2018) 76(1):75–80. 10.1684/abc.2017.1312 29336321

[103] Fessahaye G Elhassan AM Elamin EM Adam AAM Ghebremedhin A Ibrahim ME. Association of Epstein - Barr virus and breast cancer in Eritrea. Infect Agents Cancer (2017) 12:62. 10.1186/s13027-017-0173-2 PMC5740847 29299053

[104] El-Naby NEH Hassan Mohamed H Mohamed Goda A El Sayed Mohamed A. Epstein-Barr virus infection and breast invasive ductal carcinoma in Egyptian women: A single center experience. J Egypt Natl Canc Inst (2017) 29(2):77–82. 10.1016/j.jnci.2017.02.002 28462850

[105] Yahia ZA Adam AA Elgizouli M Hussein A Masri MA Kamal M. Epstein Barr virus: a prime candidate of breast cancer aetiology in Sudanese patients. Infect Agent Cancer (2014) 9(1):9. 10.1186/1750-9378-9-9 24607238 PMC3975647

[106] Rochford R Korir A Newton R. Viral-associated malignancies in Africa: are viruses ‘infectious traces’ or ‘dominant drivers’? Curr Opin Virol (2016) 20:28–33. 10.1016/j.coviro.2016.08.002 27551983

[107] Boccardo E Villa LL. Viral origins of human cancer. Curr Med Chem (2007) 14(24):2526–39. 10.2174/092986707782023316 17979705

[108] Harford JB. Viral infections and human cancers: the legacy of Denis Burkitt. Br J Haematol (2012) 156(6):709–18. 10.1111/j.1365-2141.2011.09017.x 22233526

[109] Lawson JS Salmons B Glenn WK. Oncogenic Viruses and Breast Cancer: Mouse Mammary Tumor Virus (MMTV), Bovine Leukemia Virus (BLV), Human Papilloma Virus (HPV), and Epstein-Barr Virus (EBV). Front Oncol (2018) 8:1:1. 10.3389/fonc.2018.00001 29404275 PMC5786831

[110] Ibrahim MH Rashed RA Hassan NM Al-Azhary NM Salama AI Mostafa MN. Association of cytochrome P450-1B1 gene polymorphisms with risk of breast cancer: an Egyptian study. Asian Pac J Cancer Prev (2016) 17(6):2861–6. 27356703

[111] Okobia MN Bunker CH Garte SJ Zmuda JM Ezeome ER Anyanwu SN. Cytochrome P450 1B1 Val432Leu polymorphism and breast cancer risk in Nigerian women: a case control study. Infect Agents Cancer (2009) 4 Suppl 1:S12. 10.1186/1750-9378-4-S1-S12 PMC2638457 19208203

[112] van der Merwe N Bouwens CS Pienaar R van der Merwe L Yako YY Geiger DH. CYP2D6 genotyping and use of antidepressants in breast cancer patients: test development for clinical application. Metab Brain Dis (2012) 27(3):319–26. 10.1007/s11011-012-9312-z PMC3505529 22638694

[113] Imene A Maurice AJ Arij M Sofia P Saad S. Breast cancer association with CYP1A2 activity and gene polymorphisms–a preliminary case-control study in tunisia. Asian Pac J Cancer Prev (2015) 16(8):3559–63. 10.7314/apjcp.2015.16.8.3559 25921178

[114] Khedhaier A Remadi S Corbex M Ahmed SB Bouaouina N Mestiri S. Glutathione S-transferases (GSTT1 and GSTM1) gene deletions in Tunisians: susceptibility and prognostic implications in breast carcinoma. Br J Cancer (2003) 89(8):1502–7. 10.1038/sj.bjc.6601292 PMC2394332 14562023

[115] Adegoke OJ Shu XO Gao YT Cai Q Breyer J Smith J. Genetic polymorphisms in uridine diphospho-glucuronosyltransferase 1A1 (UGT1A1) and risk of breast cancer. Breast Cancer Res Treat (2004) 85(3):239–45. 10.1023/B:BREA.0000025419.26423.b8 15111762

[116] Huo D Kim HJ Adebamowo CA Ogundiran TO Akang EE Campbell O. Genetic polymorphisms in uridine diphospho-glucuronosyltransferase 1A1 and breast cancer risk in Africans. Breast Cancer Res Treat (2008) 110(2):367–76. 10.1007/s10549-007-9720-7 PMC4384416 17909964

[117] Habyarimana T Bakri Y Mugenzi P Mazarati JB Attaleb M El Mzibri M. Association between glutathione peroxidase 1 codon 198 variant and the occurrence of breast cancer in Rwanda. Mol Genet Genomic Med (2018) 6(2):268–75. 10.1002/mgg3.367 PMC5902397 29411539

[118] Fawzy MS Aly NM Shalaby SM El-Sawy WH Abdul-Maksoud RS. Cyclooxygenase-2 169C>G and 8473T>C gene polymorphisms and prostaglandin E2 level in breast cancer: a case-control study. Gene (2013) 527(2):601–5. 10.1016/j.gene.2013.06.007 23792017

[119] Mohammad MA Zeeneldin AA Abd Elmageed ZY Khalil EH Mahdy SM Sharada HM. Clinical relevance of cyclooxygenase-2 and matrix metalloproteinases (MMP-2 and MT1-MMP) in human breast cancer tissue. Mol Cell Biochem (2012) 366(1-2):269–75. 10.1007/s11010-012-1305-z 22527932

[120] Hussein YM Gharib AF Etewa RL ElSawy WH. Association of L55M and Q192R polymorphisms in paraoxonase 1 (PON1) gene with breast cancer risk and their clinical significance. Mol Cell Biochem (2011) 351(1-2):117–23. 10.1007/s11010-011-0718-4 21229382

[121] Mestiri S Bouaouina N Ben Ahmed S Chouchane L. A functional polymorphism of the tumor necrosis factor receptor-II gene associated with the survival and relapse prediction of breast carcinoma. Cytokine (2005) 30(4):182–7. 10.1016/j.cyto.2005.01.007 15863392

[122] Mestiri S Bouaouina N Ahmed SB Khedhaier A Jrad BB Remadi S. Genetic variation in the tumor necrosis factor-alpha promoter region and in the stress protein hsp70-2: susceptibility and prognostic implications in breast carcinoma. Cancer (2001) 91(4):672–8. 10.1002/1097-0142(20010215)91:4<672::aid-cncr1050>3.0.co;2-j 11241233

[123] Mahfoudh W Bouaouina N Gabbouj S Chouchane L. FASL-844 T/C polymorphism: a biomarker of good prognosis of breast cancer in the Tunisian population. Hum Immunol (2012) 73(9):932–8. 10.1016/j.humimm.2012.06.001 22732091

[124] Zidi I Dziri O Zidi N Sebai R Boujelebene N Ben Hassine A. Association of HLA-G +3142 C>G polymorphism and breast cancer in Tunisian population. Immunol Res (2016) 64(4):961–8. 10.1007/s12026-015-8782-6 26754763

[125] Baccar Harrath A Yacoubi Loueslati B Troudi W Hmida S Sedkaoui S Dridi A. HLA class II polymorphism: protective or risk factors to breast cancer in Tunisia? Pathol Oncol Res (2006) 12(2):79–81. 10.1007/bf02893448 16799707

[126] Soliman AS Kleer CG Mrad K Karkouri M Omar S Khaled HM. Inflammatory breast cancer in north Africa: comparison of clinical and molecular epidemiologic characteristics of patients from Egypt, Tunisia, and Morocco. Breast Dis (2011) 33(4):159–69. 10.3233/BD-2012-000337 PMC4291062 23001584

[127] Snoussi K Mahfoudh W Bouaouina N Fekih M Khairi H Helal AN. Combined effects of IL-8 and CXCR2 gene polymorphisms on breast cancer susceptibility and aggressiveness. BMC Cancer (2010) 10:283. 10.1186/1471-2407-10-283 20540789 PMC2895614

[128] Snoussi K Mahfoudh W Bouaouina N Ahmed SB Helal AN Chouchane L. Genetic variation in IL-8 associated with increased risk and poor prognosis of breast carcinoma. Hum Immunol (2006) 67(1-2):13–21. 10.1016/j.humimm.2006.03.018 16698420

[129] Snoussi K Strosberg AD Bouaouina N Ben Ahmed S Chouchane L. Genetic variation in pro-inflammatory cytokines (interleukin-1beta, interleukin-1alpha and interleukin-6) associated with the aggressive forms, survival, and relapse prediction of breast carcinoma. Eur Cytokine Netw (2005) 16(4):253–60. 16464738

[130] Al Azhary NM Kamel MM Ismail YM Mahmoud AA Radwan EM. The Role of Genetic Polymorphisms in Nrf2 and P73 in Egyptian Women with Breast Cancer. Asian Pac J Cancer Prev (2016) 17(11):4945–9. 10.22034/APJCP.2016.17.11.4945 PMC5454701 28032721

[131] Welsh J. Function of the vitamin D endocrine system in mammary gland and breast cancer. Mol Cell Endocrinol (2017) 453:88–95. 10.1016/j.mce.2017.04.026 28579119 PMC5538720

[132] Hardiman G Savage SJ Hazard ES Wilson RC Courtney SM Smith MT. Systems analysis of the prostate transcriptome in African-American men compared with European-American men. Pharmacogenomics (2016) 17(10):1129–43. 10.2217/pgs-2016-0025 PMC6040053 27359067

[133] El-Shorbagy HM Mahmoud NH Sabet S. Association of vitamin D receptor gene polymorphisms with breast cancer risk in an Egyptian population. Tumour Biol (2017) 39(10):1–9. 10.1177/1010428317727738. 1010428317727738. 29022486

[134] Abd-Elsalam EA Ismaeil NA Abd-Alsalam HS. Vitamin D receptor gene polymorphisms and breast cancer risk among postmenopausal Egyptian women. Tumour Biol (2015) 36(8):6425–31. 10.1007/s13277-015-3332-3 25804799

[135] Shaker OG Senousy MA. Association of SNP-SNP interactions between RANKL, OPG, CHI3L1, and VDR genes with breast cancer risk in Egyptian women. Clin Breast Cancer (2019) 19(1):e220–38. 10.1016/j.clbc.2018.09.004 30309792

[136] Ahmed JH Makonnen E Fotoohi A Yimer G Seifu D Assefa M. Vitamin D status and association of VDR genetic polymorphism to risk of breast cancer in ethiopia. Nutrients (2019) 11(2):1–14. 10.3390/nu11020289 PMC6412905 30699973

[137] Wang S Huo D Kupfer S Alleyne D Ogundiran TO Ojengbede O. Genetic variation in the vitamin D related pathway and breast cancer risk in women of African ancestry in the root consortium. Int J Cancer (2018) 142(1):36–43. 10.1002/ijc.31038 28891071 PMC5755399

[138] Wang S Huo D Ogundiran TO Ojengbede O Zheng W Nathanson KL. Genetic variation in the Hippo pathway and breast cancer risk in women of African ancestry. Mol Carcinog (2018) 57(10):1311–8. 10.1002/mc.22845 PMC6662580 29873413

[139] El-Hussiny MA Atwa MA Rashad WE Shaheen DA Elkady NM. Leptin receptor Q223R polymorphism in Egyptian female patients with breast cancer. Contemp Oncol (Pozn) (2017) 21(1):42–7. 10.5114/wo.2017.66655 PMC5385477 28435397

[140] Okobia MN Bunker CH Garte SJ Zmuda JM Ezeome ER Anyanwu SN. Leptin receptor Gln223Arg polymorphism and breast cancer risk in Nigerian women: a case control study. BMC Cancer (2008) 8:338. 10.1186/1471-2407-8-338 19017403 PMC2613914

[141] Snoussi K Strosberg AD Bouaouina N Ben Ahmed S Helal AN Chouchane L. Leptin and leptin receptor polymorphisms are associated with increased risk and poor prognosis of breast carcinoma. BMC Cancer (2006) 6:38. 10.1186/1471-2407-6-38 16504019 PMC1397853

[142] Hosney M Sabet S El-Shinawi M Gaafar KM Mohamed MM. Leptin is overexpressed in the tumor microenvironment of obese patients with estrogen receptor positive breast cancer. Exp Ther Med (2017) 13(5):2235–46. 10.3892/etm.2017.4291 PMC5443182 28565832

[143] Habel AF Ghali RM Bouaziz H Daldoul A Hadj-Ahmed M Mokrani A. Common matrix metalloproteinase-2 gene variants and altered susceptibility to breast cancer and associated features in Tunisian women. Tumour Biol (2019) 41(4):1–8. 10.1177/1010428319845749 31014197

[144] Bawazeer S Sabry D Mahmoud RH Elhanbuli HM Yassen NN Abdelhafez MN. Association of SPARC gene polymorphisms rs3210714 and rs7719521 with VEGF expression and utility of Nottingham Prognostic Index scoring in breast cancer in a sample of Egyptian women. Mol Biol Rep (2018) 45(6):2313–24. 10.1007/s11033-018-4394-2 30259245

[145] Ben Nejima D Ben Zarkouna Y Gammoudi A Manai M Boussen H. Prognostic impact of polymorphism of matrix metalloproteinase-2 and metalloproteinase tissue inhibitor-2 promoters in breast cancer in Tunisia: case-control study. Tumour Biol (2015) 36(5):3815–22. 10.1007/s13277-014-3023-5 25656607

[146] Rahoui J Sbitti Y Touil N Laraqui A Ibrahimi A Rhrab B. The single nucleotide polymorphism +936 C/T VEGF is associated with human epidermal growth factor receptor 2 expression in Moroccan breast cancer women. Med Oncol (2014) 31(12):336. 10.1007/s12032-014-0336-6 25412937

[147] Rahoui J Laraqui A Sbitti Y Touil N Ibrahimi A Ghrab B. Investigating the association of vascular endothelial growth factor polymorphisms with breast cancer: a Moroccan case-control study. Med Oncol (2014) 31(9):193. 10.1007/s12032-014-0193-3 25148899

[148] Youssef NS Hakim SA. Association of Fascin and matrix metalloproteinase-9 expression with poor prognostic parameters in breast carcinoma of Egyptian women. Diagn Pathol (2014) 9:136. 10.1186/1746-1596-9-136 24993803 PMC4099107

[149] Kharrat N Al’Fadhli S Rebai M Aifa MS Kallel I Khabir A. (AC) dinucleotide repeat polymorphism in intron 1 of human EGFR shows ethnic specificities and high evidence for association with breast cancer. Int J Biol Markers (2007) 22(4):258–64. 10.5301/jbm.2008.1479 18161656

[150] Garner CP Ding YC John EM Ingles SA Olopade OI Huo D. Genetic variation in IGFBP2 and IGFBP5 is associated with breast cancer in populations of African descent. Hum Genet (2008) 123(3):247–55. 10.1007/s00439-008-0468-x PMC4406274 18210156

[151] Jouali F Marchoudi N Talbi S Bilal B El Khasmi M Rhaissi H. Detection of PIK3/AKT pathway in Moroccan population with triple negative breast cancer. BMC Cancer (2018) 18(1):900. 10.1186/s12885-018-4811-x 30227836 PMC6145190

[152] Hamdi Y Ben Rekaya M Jingxuan S Nagara M Messaoud O Benammar Elgaaied A. A genome wide SNP genotyping study in the Tunisian population: specific reporting on a subset of common breast cancer risk loci. BMC Cancer (2018) 18(1):1295. 10.1186/s12885-018-5133-8 30594178 PMC6310952

[153] Lupicki K Elifio-Esposito S Fonseca AS Weber SH Sugita B Langa BC. Patterns of copy number alterations in primary breast tumors of South African patients and their impact on functional cellular pathways. Int J Oncol (2018) 53(6):2745–57. 10.3892/ijo.2018.4589 30320392

[154] Shan J Mahfoudh W Dsouza SP Hassen E Bouaouina N Abdelhak S. Genome-Wide Association Studies (GWAS) breast cancer susceptibility loci in Arabs: susceptibility and prognostic implications in Tunisians. Breast Cancer Res Treat (2012) 135(3):715–24. 10.1007/s10549-012-2202-6 PMC3439608 22910930

[155] Hamdi Y Boujemaa M Ben Rekaya M Ben Hamda C Mighri N El Benna H. Family specific genetic predisposition to breast cancer: results from Tunisian whole exome sequenced breast cancer cases. J Transl Med (2018) 16(1):158. 10.1186/s12967-018-1504-9 29879995 PMC5992876

[156] Riahi A Radmanesh H Schurmann P Bogdanova N Geffers R Meddeb R. Exome sequencing and case-control analyses identify RCC1 as a candidate breast cancer susceptibility gene. Int J Cancer (2018) 142(12):2512–7. 10.1002/ijc.31273 29363114

[157] Kim YC Soliman AS Cui J Ramadan M Hablas A Abouelhoda M. Unique features of germline variation in five Egyptian familial breast cancer families revealed by exome sequencing. PloS One (2017) 12(1):e0167581. 10.1371/journal.pone.0167581 28076423 PMC5226826

[158] Belaiba F Medimegh I Ammar M Jemni F Mezlini A Romdhane KB. Expression and polymorphism of micro-RNA according to body mass index and breast cancer presentation in Tunisian patients. J Leukoc Biol (2019) 105(2):317–27. 10.1002/JLB.3VMA0618-218R 30303554

[159] Pollard J Burns PA Hughes TA Ho-Yen C Jones JL Mukherjee G. Differential expression of microRNAs in breast cancers from four different ethnicities. Pathobiology (2018) 85(4):220–6. 10.1159/000488456 29791912

[160] Zidan HE Karam RA El-Seifi OS Abd Elrahman TM. Circulating long non-coding RNA MALAT1 expression as molecular biomarker in Egyptian patients with breast cancer. Cancer Genet (2018) 220:32–7. 10.1016/j.cancergen.2017.11.005 29310836

[161] Debouki-Joudi S Trifa F Khabir A Sellami-Boudawara T Frikha M Daoud J. CpG methylation of APC promoter 1A in sporadic and familial breast cancer patients. Cancer Biomark (2017) 18(2):133–41. 10.3233/CBM-160005 PMC13020579 27983523

[162] Hamdi K Blancato J Goerlitz D Islam M Neili B Abidi A. Circulating cell-free miRNA expression and its association with clinicopathologic features in inflammatory and non- inflammatory breast cancer. Asian Pac J Cancer Prev (2016) 17(4):1801–10. 10.7314/apjcp.2016.17.4.1801 27221856

[163] Hafez MM Hassan ZK Zekri AR Gaber AA Al Rejaie SS Sayed-Ahmed MM. MicroRNAs and metastasis-related gene expression in Egyptian breast cancer patients. Asian Pac J Cancer Prev (2012) 13(2):591–8. 10.7314/apjcp.2012.13.2.591 22524830

[164] Zaki SM Abdel-Azeez HA El Nagar MR Metwally KA MM SA. Analysis of FHIT gene methylation in egyptian breast cancer women: association with clinicopathological features. Asian Pac J Cancer Prev (2015) 16(3):1235–9. 10.7314/apjcp.2015.16.3.1235 25735361

[165] Trifa F Karray-Chouayekh S Jmaa ZB Jmal E Khabir A Sellami-Boudawara T. Frequent CpG methylation of ubiquitin carboxyl-terminal hydrolase 1 (UCHL1) in sporadic and hereditary Tunisian breast cancer patients: clinical significance. Med Oncol (2013) 30(1):418. 10.1007/s12032-012-0418-2 23315218

[166] Karray-Chouayekh S Trifa F Khabir A Boujelbene N Sellami-Boudawara T Daoud J. Methylation status and overexpression of COX-2 in Tunisian patients with ductal invasive breast carcinoma. Tumour Biol (2011) 32(3):461–8. 10.1007/s13277-010-0139-0 21153458

[167] Karray-Chouayekh S Trifa F Khabir A Boujelbane N Sellami-Boudawara T Daoud J. Aberrant methylation of RASSF1A is associated with poor survival in Tunisian breast cancer patients. J Cancer Res Clin Oncol (2010) 136(2):203–10. 10.1007/s00432-009-0649-6 PMC11828106 19657672

[168] Adeloye D David RA Aderemi AV Iseolorunkanmi A Oyedokun A Iweala EE. An estimate of the incidence of prostate cancer in Africa: a systematic review and meta-analysis. PloS One (2016) 11(4):e0153496. 10.1371/journal.pone.0153496 27073921 PMC4830589

[169] Odedina FT Akinremi TO Chinegwundoh F Roberts R Yu D Reams RR. Prostate cancer disparities in Black men of African descent: a comparative literature review of prostate cancer burden among Black men in the United States, Caribbean, United Kingdom, and West Africa. Infect Agents Cancer (2009) 4 Suppl 1:S2. 10.1186/1750-9378-4-S1-S2 PMC2638461 19208207

[170] Yassin A AlRumaihi K Alzubaidi R Alkadhi S Al Ansari A. Testosterone, testosterone therapy and prostate cancer. Aging Male (2019) 22(4):219–27. 10.1080/13685538.2018.1524456 30614347

[171] Hsing AW Chu LW Stanczyk FZ. Androgen and prostate cancer: is the hypothesis dead? Cancer Epidemiol Biomarkers Prev (2008) 17(10):2525–30. 10.1158/1055-9965.EPI-08-0448 18842992

[172] Novillo A Romero-Lorca A Gaibar M Bahri R Harich N Sanchez-Cuenca D. Genetic diversity of CYP3A4 and CYP3A5 polymorphisms in North African populations from Morocco and Tunisia. Int J Biol Markers (2015) 30(1):e148–51. 10.5301/jbm.5000118 25385241

[173] Fernandez P Zeigler-Johnson CM Spangler E van der Merwe A Jalloh M Gueye SM. Androgen metabolism gene polymorphisms, associations with prostate cancer risk and pathological characteristics: a comparative analysis between South African and senegalese men. Prostate Cancer (2012) 2012:798634. 10.1155/2012/798634 23091730 PMC3468128

[174] Souiden Y Mahdouani M Chaieb K Bakhrouf A Mahdouani K. Lack of association of CYP1A1 polymorphism with prostate cancer susceptibility of Tunisian men. Genet testing Mol Biomarkers (2012) 16(7):661–6. 10.1089/gtmb.2011.0212 22304463

[175] Souiden Y Mahdouani M Chaieb K Elkamel R Mahdouani K. CYP17 gene polymorphism and prostate cancer susceptibility in a Tunisian population. Cancer Epidemiol (2011) 35(5):480–4. 10.1016/j.canep.2010.11.008 21193363

[176] Fernandez P De Beer PM Van der Merwe L Heyns CF. Genetic variations in androgen metabolism genes and associations with prostate cancer in South African men. S Afr Med J (2010) 100(11):741–5. 10.7196/samj.4104 21081028

[177] Akinloye O Gromoll J Simoni M. Variation in CAG and GGN repeat lengths and CAG/GGN haplotype in androgen receptor gene polymorphism and prostate carcinoma in Nigerians. Br J BioMed Sci (2011) 68(3):138–42. 10.1080/09674845.2011.11730341 21950206

[178] Esteban E Rodon N Via M Gonzalez-Perez E Santamaria J Dugoujon JM. and GGC polymorphisms in Mediterraneans: repeat dynamics and population relationships. J Hum Genet (2006) 51(2):129–36. 10.1007/s10038-005-0336-7 16365681

[179] Novillo A Gaibar M Romero-Lorca A Chaabani H Amir N Moral P. UDP-glucuronosyltransferase genetic variation in North African populations: a comparison with African and European data. Ann Hum Biol (2018) 45(6-8):516–23. 10.1080/03014460.2018.1559354 30616396

[180] Benabdelkrim M Djeffal O Berredjem H. GSTM1 and GSTT1 polymorphisms and susceptibility to prostate cancer: a case-control study of the algerian population. Asian Pac J Cancer Prev (2018) 19(10):2853–8. 10.22034/APJCP.2018.19.10.2853 PMC6291025 30362312

[181] Fernandez-Santander A Novillo A Gaibar M Romero-Lorca A Moral P Sanchez-Cuenca D. Cytochrome and sulfotransferase gene variation in north African populations. Pharmacogenomics (2016) 17(13):1415–23. 10.2217/pgs-2016-0016 27471773

[182] Souiden Y Mahdouani M Chaieb K Elkamel R Mahdouani K. Polymorphisms of glutathione-S-transferase M1 and T1 and prostate cancer risk in a Tunisian population. Cancer Epidemiol (2010) 34(5):598–603. 10.1016/j.canep.2010.06.002 20599479

[183] Du Z Lubmawa A Gundell S Wan P Nalukenge C Muwanga P. Genetic risk of prostate cancer in Ugandan men. Prostate (2018) 78(5):370–6. 10.1002/pros.23481 PMC7534689 29356057

[184] Cook MB Wang Z Yeboah ED Tettey Y Biritwum RB Adjei AA. A genome-wide association study of prostate cancer in West African men. Hum Genet (2014) 133(5):509–21. 10.1007/s00439-013-1387-z PMC3988225 24185611

[185] Shan J Al-Rumaihi K Rabah D Al-Bozom I Kizhakayil D Farhat K. Genome scan study of prostate cancer in Arabs: identification of three genomic regions with multiple prostate cancer susceptibility loci in Tunisians. J Transl Med (2013) 11:121. 10.1186/1479-5876-11-121 23668334 PMC3659060

[186] Jaratlerdsiri W Chan EKF Gong T Petersen DC Kalsbeek AMF Venter PA. Whole-genome sequencing reveals elevated tumor mutational burden and initiating driver mutations in African men with treatment-naive, high-risk prostate cancer. Cancer Res (2018) 78(24):6736–46. 10.1158/0008-5472.CAN-18-0254 30217929

[187] Blackburn J Vecchiarelli S Heyer EE Patrick SM Lyons RJ Jaratlerdsiri W. TMPRSS2-ERG fusions linked to prostate cancer racial health disparities: A focus on Africa. Prostate (2019) 79(10):1191–6. 10.1002/pros.23823 PMC6617820 31090091

[188] Wang Y Freedman JA Liu H Moorman PG Hyslop T George DJ. Associations between RNA splicing regulatory variants of stemness-related genes and racial disparities in susceptibility to prostate cancer. Int J Cancer (2017) 141(4):731–43. 10.1002/ijc.30787 PMC5512873 28510291

[189] Abdel-Hady A El-Hindawi A Hammam O Khalil H Diab S El-Aziz SA. Expression of ERG Protein and TMRPSS2-ERG Fusion in Prostatic Carcinoma in Egyptian Patients. Open Access Maced J Med Sci (2017) 5(2):147–54. 10.3889/oamjms.2017.037 PMC5420765 28507619

[190] Villar S Le Roux-Goglin E Gouas DA Plymoth A Ferro G Boniol M. Seasonal variation in TP53 R249S-mutated serum DNA with aflatoxin exposure and hepatitis B virus infection. Environ Health Perspect (2011) 119(11):1635–40. 10.1289/ehp.1103539 PMC3226502 21768053

[191] Hsia CC Kleiner DE Jr. Axiotis CA Di Bisceglie A Nomura AM Stemmermann GN. Mutations of p53 gene in hepatocellular carcinoma: roles of hepatitis B virus and aflatoxin contamination in the diet. J Natl Cancer Inst (1992) 84(21):1638–41. 10.1093/jnci/84.21.1638 1279184

[192] Ezzikouri S Essaid El Feydi A Afifi R Benazzouz M Hassar M Pineau P. Impact of TP53 codon 72 and MDM2 promoter 309 allelic dosage in a Moroccan population with hepatocellular carcinoma. Int J Biol Markers (2011) 26(4):229–33. 10.5301/JBM.2011.8881 22180176

[193] Ezzikouri S El Feydi AE Chafik A Benazzouz M El Kihal L Afifi R. The Pro variant of the p53 codon 72 polymorphism is associated with hepatocellular carcinoma in Moroccan population. Hepatol Res (2007) 37(9):748–54. 10.1111/j.1872-034X.2007.00126.x 17573955

[194] El Far MA Atwa MA Yahya RS El Basuni MA. Evaluation of serum levels of p53 in hepatocellular carcinoma in Egypt. Clin Chem Lab Med (2006) 44(5):653–6. 10.1515/CCLM.2006.091 16681440

[195] El-Kafrawy SA Abdel-Hamid M El-Daly M Nada O Ismail A Ezzat S. P53 mutations in hepatocellular carcinoma patients in Egypt. Int J Hyg Environ Health (2005) 208(4):263–70. 10.1016/j.ijheh.2005.02.002 16078640

[196] Martins C Kedda MA Kew MC. Characterization of six tumor suppressor genes and microsatellite instability in hepatocellular carcinoma in southern African blacks. World J Gastroenterol (1999) 5(6):470–6. 10.3748/wjg.v5.i6.470 PMC4688788 11819494

[197] Coursaget P Depril N Chabaud M Nandi R Mayelo V LeCann P. High prevalence of mutations at codon 249 of the p53 gene in hepatocellular carcinomas from Senegal. Br J Cancer (1993) 67(6):1395–7. 10.1038/bjc.1993.258 PMC1968506 8390289

[198] Anonymous. AGG—-AGT mutation in the codon number 249 of p53 gene is the frequent cause of liver cancers in China and South Africa. Indian J Exp Biol (1991) 29(8):798–9. 1769728

[199] Igetei R Otegbayo JA Ndububa DA Lesi OA Anumudu CI Hainaut P. Detection of p53 codon 249 mutation in Nigerian patients with hepatocellular carcinoma using a novel evaluation of cell-free DNA. Ann Hepatol (2008) 7(4):339–44. 10.1016/S1665-2681(19)31834-4 19034233

[200] Kirk GD Camus-Randon AM Mendy M Goedert JJ Merle P Trepo C. Ser-249 p53 mutations in plasma DNA of patients with hepatocellular carcinoma from The Gambia. J Natl Cancer Inst (2000) 92(2):148–53. 10.1093/jnci/92.2.148 10639517

[201] Kimbi GC Kew MC Yu MC Arakawa K Hodkinson J. 249ser p53 mutation in the serum of black southern African patients with hepatocellular carcinoma. J Gastroenterol Hepatol (2005) 20(8):1185–90. 10.1111/j.1440-1746.2005.03951.x 16048565

[202] Kirby GM Batist G Fotouhi-Ardakani N Nakazawa H Yamasaki H Kew M. Allele-specific PCR analysis of p53 codon 249 AGT transversion in liver tissues from patients with viral hepatitis. Int J Cancer (1996) 68(1):21–5. 10.1002/(sici)1097-0215(19960927)68:1<21::Aid-ijc4>3.0.Co;2-z 8895534

[203] Marchio A Amougou Atsama M Bere A Komas NP Noah Noah D Atangana PJA. Droplet digital PCR detects high rate of TP53 R249S mutants in cell-free DNA of middle African patients with hepatocellular carcinoma. Clin Exp Med (2018) 18(3):421–31. 10.1007/s10238-018-0502-9 29749584

[204] Ndububa DA Yakicier CM Ojo OS Adeodu OO Rotimi O Ogunbiyi O. P53 codon 249 mutation in hepatocellular carcinomas from Nigeria. Afr J Med Med Sci (2001) 30(1-2):125–7. 14510167

[205] Szymanska K Lesi OA Kirk GD Sam O Taniere P Scoazec JY. Ser-249TP53 mutation in tumour and plasma DNA of hepatocellular carcinoma patients from a high incidence area in the Gambia, West Africa. Int J Cancer (2004) 110(3):374–9. 10.1002/ijc.20103 15095302

[206] Yap EP Cooper K Maharaj B McGee JO. p53 codon 249ser hot-spot mutation in HBV-negative hepatocellular carcinoma. Lancet (1993) 341(8839):251. 10.1016/0140-6736(93)90124-y 8093542

[207] Wahab AH Kassem AM Matter S El Deen AF Helmy AS Ismaeil MM. Role of KLF6 tumor suppressor gene mutations in the development of colorectal carcinoma in an Egyptian population. Hepatogastroenterology (2010) 57(104):1405–10. 21443094

[208] Lin Y Shi CY Li B Soo BH Mohammed-Ali S Wee A. Tumour suppressor p53 and Rb genes in human hepatocellular carcinoma. Ann Acad Med Singapore (1996) 25(1):22–30. 8779541

[209] Rebbani K Ezzikouri S Marchio A Kandil M Pineau P Benjelloun S. MDM2 285G>C and 344T>A gene variants and their association with hepatocellular carcinoma: a Moroccan case-control study. Infect Agents Cancer (2014) 9(1):11. 10.1186/1750-9378-9-11 PMC3986458 24708820

[210] Ezzikouri S El Feydi AE Afifi R El Kihal L Benazzouz M Hassar M. MDM2 SNP309T>G polymorphism and risk of hepatocellular carcinoma: a case-control analysis in a Moroccan population. Cancer Detect Prev (2009) 32(5-6):380–5. 10.1016/j.cdp.2009.01.003 19233569

[211] Pineau P Ezzikouri S Marchio A Benazzouz M Cordina E Afifi R. Genomic stability prevails in North-African hepatocellular carcinomas. Dig Liver Dis (2007) 39(7):671–7. 10.1016/j.dld.2007.03.012 17531558

[212] Paruk IM Pirie FJ Motala AA. Non-alcoholic fatty liver disease in Africa: a hidden danger. Glob Health Epidemiol Genom (2019) 4:e3. 10.1017/gheg.2019.2 31019719 PMC6465678

[213] Tharwat E Gad GFM Nazmy MH Mohamed HI Hamza N Wahid A. Impact of IL-27p28 (rs153109) and TNF-alpha (rs1800629) Genetic polymorphisms on the progression of HCV infection in Egyptian patients. Immunol Invest (2019) 48(3):255–67. 10.1080/08820139.2018.1510958 30204505

[214] ElSheshtawy NM Nour MS Hefny Z Samir A. Gene polymorphisms of Interleukin 1? and Metalloproteinase 3 in Hepatitis C Infected Patients and Hepatocellular Carcinoma Patients. Egypt J Immunol (2017) 24(1):1–8. 29120572

[215] Sghaier I Mouelhi L Rabia NA Alsaleh BR Ghazoueni E Almawi WY. Genetic variants in IL-6 and IL-10 genes and susceptibility to hepatocellular carcinoma in HCV infected patients. Cytokine (2017) 89:62–7. 10.1016/j.cyto.2016.10.004 28340949

[216] Elsayed HM Nabiel Y Sheta T. IL12 Gene Polymorphism in association with hepatocellular carcinoma in HCV-infected Egyptian patients. Immunol Invest (2017) 46(2):123–33. 10.1080/08820139.2016.1229789 27819525

[217] Ma EL Abd El Fatah G Zaghla H. IL17A gene polymorphism, serum IL17 and total IgE in Egyptian population with chronic HCV and hepatocellular carcinoma. Immunol Lett (2015) 168(2):240–5. 10.1016/j.imlet.2015.09.004 26367076

[218] Labib HA Ahmed HS Shalaby SM Wahab EA Hamed EF. Genetic polymorphism of IL-23R influences susceptibility to HCV-related hepatocellular carcinoma. Cell Immunol (2015) 294(1):21–4. 10.1016/j.cellimm.2015.01.012 25666505

[219] Talaat RM Esmail AA Elwakil R Gurgis AA Nasr MI. Tumor necrosis factor-alpha -308G/A polymorphism and risk of hepatocellular carcinoma in hepatitis C virus-infected patients. Chin J Cancer (2012) 31(1):29–35. 10.5732/cjc.011.10258 22200181 PMC3777466

[220] Mamdouh S Khorshed F Aboushousha T Hamdy H Diab A Seleem M. Evaluation of Mir-224, Mir-215 and Mir-143 as serum biomarkers for HCV associated hepatocellular carcinoma. Asian Pac J Cancer Prev (2017) 18(11):3167–71. 10.22034/APJCP.2017.18.11.3167 PMC5773807 29172295

[221] El-Abd NE Fawzy NA El-Sheikh SM Soliman ME. Circulating miRNA-122, miRNA-199a, and miRNA-16 as biomarkers for early detection of hepatocellular carcinoma in Egyptian patients with chronic hepatitis C virus infection. Mol Diagn Ther (2015) 19(4):213–20. 10.1007/s40291-015-0148-1 26133725

[222] Motawi TM Sadik NA Shaker OG Ghaleb MH. Elevated serum microRNA-122/222 levels are potential diagnostic biomarkers in Egyptian patients with chronic hepatitis C but not hepatic cancer. Tumour Biol (2016) 37(7):9865–74. 10.1007/s13277-016-4884-6 26812693

[223] Motawi TMK El-Maraghy SA Sabry D Mehana NA. The expression of long non coding RNA genes is associated with expression with polymorphisms of HULC rs7763881 and MALAT1 rs619586 in hepatocellular carcinoma and HBV Egyptian patients. J Cell Biochem (2019) 120(9):14645–56. 10.1002/jcb.28726 31009106

[224] Darwish WS Ikenaka Y Nakayama SM Ishizuka M. An overview on mycotoxin contamination of foods in Africa. J Vet Med Sci (2014) 76(6):789–97. 10.1292/jvms.13-0563 PMC4108760 24572628

[225] Hainaut P Boyle P. Curbing the liver cancer epidemic in Africa. Lancet (2008) 371(9610):367–8. 10.1016/S0140-6736(08)60181-6 18242399

[226] Mupunga I Mngqawa P Katerere DR. Peanuts, Aflatoxins and undernutrition in children in sub-saharan Africa. Nutrients (2017) 9(12):1287. 10.3390/nu9121287 PMC5748738 29186859

[227] Rebbeck TR. Molecular epidemiology of the human glutathione S-transferase genotypes GSTM1 and GSTT1 in cancer susceptibility. Cancer Epidemiol Biomarkers Prev (1997) 6(9):733–43. 9298582

[228] Abd El-Moneim E Younis FA Allam N Gameel K Osman M. Gene deletion of glutathione S-transferase M1 and T1 and risk factors of hepatocellular carcinoma in Egyptian patients. Egypt J Immunol (2008) 15(2):125–34. 20306695

[229] Tiemersma EW Omer RE Bunschoten A van’t Veer P Kok FJ Idris MO. Role of genetic polymorphism of glutathione-S-transferase T1 and microsomal epoxide hydrolase in aflatoxin-associated hepatocellular carcinoma. Cancer Epidemiol Biomarkers Prev (2001) 10(7):785–91. 11440964

[230] El Samanoudy A Monir R Badawy A Ibrahim L Farag K El Baz S. Matrix metalloproteinase-9 gene polymorphism in hepatocellular carcinoma patients with hepatitis B and C viruses. Genet Mol Res (2014) 13(3):8025–34. 10.4238/2014.September.29.15 25299117

[231] Fakhry AB Ahmed AI AbdelAlim MA Ramadan DI. RECK gene promoter rs10814325 polymorphism in Egyptian patients with hepatocellular carcinoma on top of chronic hepatitis C viral infection. Asian Pac J Cancer Prev (2016) 17(5):2383–8. 27268601

[232] Jedy-Agba E Joko WY Liu B Buziba NG Borok M Korir A. Trends in cervical cancer incidence in sub-Saharan Africa. Br J Cancer (2020) 123(1):148–54. 10.1038/s41416-020-0831-9 PMC7341858 32336751

[233] Parkin DM Hammerl L Ferlay J Kantelhardt EJ. Cancer in Africa 2018: The role of infections. Int J Cancer (2020) 146(8):2089–103. 10.1002/ijc.32538 31254479

[234] Mboumba Bouassa RS Prazuck T Lethu T Jenabian MA Meye JF Belec L. Cervical cancer in sub-Saharan Africa: a preventable noncommunicable disease. Expert Rev Anti Infect Ther (2017) 15(6):613–27. 10.1080/14787210.2017.1322902 28440679

[235] Mboumba Bouassa RS Prazuck T Lethu T Meye JF Belec L. Cervical cancer in sub-Saharan Africa: an emerging and preventable disease associated with oncogenic human papillomavirus. Med Sante Trop (2017) 27(1):16–22. 10.1684/mst.2017.0648 28406406

[236] McKay J Tenet V Franceschi S Chabrier A Gheit T Gaborieau V. Immuno-related polymorphisms and cervical cancer risk: The IARC multicentric case-control study. PloS One (2017) 12(5):e0177775. 10.1371/journal.pone.0177775 28505207 PMC5432183

[237] Chattopadhyay K. A comprehensive review on host genetic susceptibility to human papillomavirus infection and progression to cervical cancer. Indian J Hum Genet (2011) 17(3):132–44. 10.4103/0971-6866.92087 PMC3276980 22345983

[238] de Araujo Souza PS Villa LL. Genetic susceptibility to infection with human papillomavirus and development of cervical cancer in women in Brazil. Mutat Res (2003) 544(2-3):375–83. 10.1016/j.mrrev.2003.06.013 14644340

[239] Lahsen AO Baba H Bensghir R Fayssel N Sodqi M Marih L. TP53 R72P polymorphism and susceptibility to human papillomavirus infection among women with human immunodeficiency virus in morocco: a case-control study. J Cancer Prev (2017) 22(4):248–53. 10.15430/JCP.2017.22.4.248 PMC5751843 29302583

[240] Ndiaye R Dem A Mbaye PM Gueye PM Diop G Diop PA. [Study of codon 72 of p53 gene as a risk-factor in cervical cancer in Senegal]. Bull Cancer (2014) 101(9):789–94. 10.1684/bdc.2014.1911 25025520

[241] Eltahir HA Elhassan AM Ibrahim ME. Contribution of retinoblastoma LOH and the p53 Arg/Pro polymorphism to cervical cancer. Mol Med Rep (2012) 6(3):473–6. 10.3892/mmr.2012.942 PMC3493075 22692183

[242] El khair MM Ennaji MM El kebbaj R Mhand RA Attaleb M El Mzibri M. p53 codon 72 polymorphism and risk of cervical carcinoma in Moroccan women. Med Oncol (2010) 27(3):861–6. 10.1007/s12032-009-9297-6 19771536

[243] Feng Q Hawes SE Stern JE Dem A Sow PS Dembele B. Promoter hypermethylation of tumor suppressor genes in urine from patients with cervical neoplasia. Cancer Epidemiol Biomarkers Prev (2007) 16(6):1178–84. 10.1158/1055-9965.EPI-06-0694 17548682

[244] Govan VA Loubser S Saleh D Hoffman M Williamson AL. No relationship observed between human p53 codon-72 genotype and HPV-associated cervical cancer in a population group with a low arginine-72 allele frequency. Int J Immunogenet (2007) 34(3):213–7. 10.1111/j.1744-313X.2007.00678.x 17504512

[245] Feng Q Balasubramanian A Hawes SE Toure P Sow PS Dem A. Detection of hypermethylated genes in women with and without cervical neoplasia. J Natl Cancer Inst (2005) 97(4):273–82. 10.1093/jnci/dji041 15713962

[246] Arbel-Alon S Menczer J Feldman N Glezerman M Yeremin L Friedman E. Codon 72 polymorphism of p53 in Israeli Jewish cervical cancer patients and healthy women. Int J Gynecol Cancer (2002) 12(6):741–4. 10.1046/j.1525-1438.2002.01124.x 12445252

[247] Tan SC Ankathil R. Genetic susceptibility to cervical cancer: role of common polymorphisms in apoptosis-related genes. Tumour Biol (2015) 36(9):6633–44. 10.1007/s13277-015-3868-2 26242271

[248] Zidi S Stayoussef M Alsaleh BL Gazouani E Mezlini A Ebrahim BH. Relationships between common and novel interleukin-6 gene polymorphisms and risk of cervical cancer: a case-control study. Pathol Oncol Res (2017) 23(2):385–92. 10.1007/s12253-016-0127-9 27722983

[249] Zidi S Gazouani E Stayoussef M Mezlini A Ahmed SK Yacoubi-Loueslati B. IL-10 gene promoter and intron polymorphisms as genetic biomarkers of cervical cancer susceptibility among Tunisians. Cytokine (2015) 76(2):343–7. 10.1016/j.cyto.2015.05.028 26076679

[250] Govan VA Constant D Hoffman M Williamson AL. The allelic distribution of -308 Tumor Necrosis Factor-alpha gene polymorphism in South African women with cervical cancer and control women. BMC Cancer (2006) 6:24. 10.1186/1471-2407-6-24 16438713 PMC1397852

[251] Chattopadhyay K Williamson AL Hazra A Dandara C. The combined risks of reduced or increased function variants in cell death pathway genes differentially influence cervical cancer risk and herpes simplex virus type 2 infection among black Africans and the Mixed Ancestry population of South Africa. BMC Cancer (2015) 15:680. 10.1186/s12885-015-1678-y 26458812 PMC4603903

[252] Miladi-Abdennadher I Amouri A Ayadi L Khabir A Ellouze S Tahri N. A novel pathogenic germline mutation in the adenomatous polyposis coli gene in a Tunisian family with FAP. Fam Cancer (2011) 10(3):567–71. 10.1007/s10689-011-9451-0 21598003

[253] Ibirogba SB Algar U Goldberg PA Duffield M Vorster A Ramesar R. Clinical and pathological features of hereditary mixed polyposis syndrome: report on a South African family. S Afr J Surg (2008) 46(3):90–2. 18807306

[254] Grobbelaar JJ Wilken E de Ravel TJ Nicholson DL Kotze MJ. Familial adenomatous polyposis in two Black South African families. Clin Genet (2002) 61(3):214–7. 10.1034/j.1399-0004.2002.610308.x 12000364

[255] Ramesar RS Madden MV Felix R Harocopos CJ Westbrook CA Jones G. Molecular genetics improves the management of hereditary non-polyposis colorectal cancer. S Afr Med J (2000) 90(7):709–14. 10985134

[256] Elsaid A Elshazli R El-Tarapely F Darwish H Abdel-Malak C. Association of monoallelic MUTYH mutation among Egyptian patients with colorectal cancer. Fam Cancer (2017) 16(1):83–90. 10.1007/s10689-016-9927-z 27631816

[257] Laarabi FZ Cherkaoui Jaouad I Baert-Desurmont S Ouldim K Ibrahimi A Kanouni N. The first mutations in the MYH gene reported in Moroccan colon cancer patients. Gene (2012) 496(1):55–8. 10.1016/j.gene.2011.12.024 22266422

[258] Laarabi FZ Cherkaoui Jaouad I Benazzouz A Squalli D Sefiani A. Prevalence of MYH-associated polyposis related to three recurrent mutations in Morocco. Ann Hum Biol (2011) 38(3):360–3. 10.3109/03014460.2010.521520 20939750

[259] Moussa SA Moussa A Kourda N Mezlini A Abdelli N Zerimech F. Lynch syndrome in Tunisia: first description of clinical features and germline mutations. Int J Colorectal Dis (2011) 26(4):455–67. 10.1007/s00384-010-1129-9 21311894

[260] Hitchins MP Owens SE Kwok CT Godsmark G Algar UF Ramesar RS. Identification of new cases of early-onset colorectal cancer with an MLH1 epimutation in an ethnically diverse South African cohort. Clin Genet (2011) 80(5):428–34. 10.1111/j.1399-0004.2011.01660.x 21375527

[261] Moufid FZ Bouguenouch L El Bouchikhi I Chbani L Iraqui Houssaini M Sekal M. The first molecular screening of MLH1 and MSH2 genes in moroccan colorectal cancer patients shows a relatively high mutational prevalence. Genet Test Mol Biomarkers (2018) 22(8):492–7. 10.1089/gtmb.2018.0067 30044143

[262] Abdelmaksoud-Dammak R Miladi-Abdennadher I Amouri A Tahri N Ayadi L Khabir A. High prevalence of the c.1227_1228dup (p.Glu410GlyfsX43) mutation in Tunisian families affected with MUTYH-associated-polyposis. Fam Cancer (2012) 11(3):503–8. 10.1007/s10689-012-9543-5 22744763

[263] Bougatef K Marrakchi R Kourda N Ben Lahely YB Jileni SB El Khil HK. Somatic mutation of MutYH in Tunisian patients with sporadic colorectal cancer. J Clin Lab Anal (2007) 21(6):372–4. 10.1002/jcla.20198 PMC6649000 18022921

[264] Ziada-Bouchaar H Sifi K Filali T Hammada T Satta D Abadi N. First description of mutational analysis of MLH1, MSH2 and MSH6 in Algerian families with suspected Lynch syndrome. Fam Cancer (2017) 16(1):57–66. 10.1007/s10689-016-9917-1 27468915

[265] Aissi-Ben Moussa S Moussa A Lovecchio T Kourda N Najjar T Ben Jilani S. Identification and characterization of a novel MLH1 genomic rearrangement as the cause of HNPCC in a Tunisian family: evidence for a homologous Alu-mediated recombination. Fam Cancer (2009) 8(2):119–26. 10.1007/s10689-008-9215-7 18792805

[266] Moufid FZ Bouguenouch L El Bouchikhi I Houssaini MI Ouldim K. Molecular and presymptomatic analysis of a Moroccan Lynch syndrome family revealed a novel frameshift MLH1 germline mutation. Turk J Gastroenterol (2018) 29(6):701–4. 10.5152/tjg.2018.17761 PMC6284679 30289396

[267] El-Serafi MM Bahnassy AA Ali NM Eid SM Kamel MM Abdel-Hamid NA. The prognostic value of c-Kit, K-ras codon 12, and p53 codon 72 mutations in Egyptian patients with stage II colorectal cancer. Cancer (2010) 116(21):4954–64. 10.1002/cncr.25417 20652953

[268] Karim B Florence C Kamel R Nadia K Ines O Raja M. KRAS mutation detection in Tunisian sporadic coloractal cancer patients with direct sequencing, high resolution melting and denaturating high performance liquid chromatography. Cancer Biomark (2010) 8(6):331–40. 10.3233/CBM-2011-0222 PMC13015940 22072121

[269] Ines C Donia O Rahma B Ben Ammar A Sameh A Khalfallah T. Implication of K-ras and p53 in colorectal cancer carcinogenesis in Tunisian population cohort. Tumour Biol (2014) 35(7):7163–75. 10.1007/s13277-014-1874-4 24763823

[270] Marchoudi N Amrani Hassani Joutei H Jouali F Fekkak J Rhaissi H. Distribution of KRAS and BRAF mutations in Moroccan patients with advanced colorectal cancer. Pathol Biol (Paris) (2013) 61(6):273–6. 10.1016/j.patbio.2013.05.004 23849768

[271] Raskin L Dakubo JC Palaski N Greenson JK Gruber SB. Distinct molecular features of colorectal cancer in Ghana. Cancer Epidemiol (2013) 37(5):556–61. 10.1016/j.canep.2013.07.007 PMC4267222 23962701

[272] Sammoud S Khiari M Semeh A Amine L Ines C Amira A. Relationship between expression of ras p21 oncoprotein and mutation status of the K-ras gene in sporadic colorectal cancer patients in Tunisia. Appl Immunohistochem Mol Morphol (2012) 20(2):146–52. 10.1097/PAI.0b013e3182240de1 21768877

[273] Abdulkareem FB Sanni LA Richman SD Chambers P Hemmings G Grabsch H. KRAS and BRAF mutations in Nigerian colorectal cancers. West Afr J Med (2012) 31(3):198–203. 23310942

[274] Aissi S Buisine MP Zerimech F Kourda N Moussa A Manai M. TP53 mutations in colorectal cancer from Tunisia: relationships with site of tumor origin, microsatellite instability and KRAS mutations. Mol Biol Rep (2014) 41(3):1807–13. 10.1007/s11033-014-3030-z 24443225

[275] Bennani B Gilles S Fina F Nanni I Ibrahimi SA Riffi AA. Mutation analysis of BRAF exon 15 and KRAS codons 12 and 13 in Moroccan patients with colorectal cancer. Int J Biol Markers (2010) 25(4):179–84. 10.5301/JBM.2010.6091 21161938

[276] Irabor DO Oluwasola OA Ogunbiyi OJ Ogun OG Okolo CA Melas M. Microsatellite Instability Is Common in Colorectal Cancer in Native Nigerians. Anticancer Res (2017) 37(5):2649–54. 10.21873/anticanres.11612 28476840

[277] Pyatt R Chadwick RB Johnson CK Adebamowo C de la Chapelle A Prior TW. Polymorphic variation at the BAT-25 and BAT-26 loci in individuals of African origin. Implications for microsatellite instability testing. Am J Pathol (1999) 155(2):349–53. 10.1016/S0002-9440(10)65131-0 PMC1866867 10433928

[278] Sekal M Ameurtesse H Chbani L Ouldim K Bennis S Abkari M. Epigenetics could explain some Moroccan population colorectal cancers peculiarities: microsatellite instability pathway exploration. Diagn Pathol (2015) 10:77. 10.1186/s13000-015-0326-9 26104511 PMC4477595

[279] Naidoo R Tarin M Chetty R. A comparative microsatellite analysis of colorectal cancer in patients <35 years and >50 years of age. Am J Gastroenterol (2000) 95(11):3266–75. 10.1111/j.1572-0241.2000.03208.x 11095352

[280] Kria Ben Mahmoud L Arfaoui A Khiari M Chaar I Lounis A Sammoud S. Evaluation of microsatellite instability, MLH1 expression and hMLH1 promoter hypermethylation in colorectal carcinomas among Tunisians patients. Tunis Med (2012) 90(8-9):646–53. 22987381

[281] Ziadi S Ksiaa F Ben Gacem R Labaied N Mokni M Trimeche M. Clinicopathologic characteristics of colorectal cancer with microsatellite instability. Pathol Res Pract (2014) 210(2):98–104. 10.1016/j.prp.2013.10.004 24286815

[282] Abdelmaksoud-Dammak R Saadallah-Kallel A Miladi-Abdennadher I Ayedi L Khabir A Sallemi-Boudawara T. CpG methylation of ubiquitin carboxyl-terminal hydrolase 1 (UCHL1) and P53 mutation pattern in sporadic colorectal cancer. Tumour Biol (2016) 37(2):1707–14. 10.1007/s13277-015-3902-4 26314856

[283] Chaar I Amara S Elamine OE Khiari M Ounissi D Khalfallah T. Biological significance of promoter hypermethylation of p14/ARF gene: relationships to p53 mutational status in Tunisian population with colorectal carcinoma. Tumour Biol J Int Soc Oncodevelopmental Biol Med (2014) 35(2):1439–49. 10.1007/s13277-013-1198-9 PMC3932170 24065196

[284] Nieminen TT Shoman S Eissa S Peltomaki P Abdel-Rahman WM. Distinct genetic and epigenetic signatures of colorectal cancers according to ethnic origin. Cancer Epidemiol Biomarkers Prev (2012) 21(1):202–11. 10.1158/1055-9965.EPI-11-0662 22028395

[285] Chan AO Soliman AS Zhang Q Rashid A Bedeir A Houlihan PS. Differing DNA methylation patterns and gene mutation frequencies in colorectal carcinomas from Middle Eastern countries. Clin Cancer Res (2005) 11(23):8281–7. 10.1158/1078-0432.Ccr-05-1000 16322286

[286] Chaar I Amara S Khiari M Ounissi D Dhraif M Ben Hamida AE. Relationship between MDM2 and p53 alterations in colorectal cancer and their involvement and prognostic value in the Tunisian population. Appl Immunohistochem Mol Morphol (2013) 21(3):228–36. 10.1097/PAI.0b013e31825f4e20 22914606

[287] Arfaoui AT Kriaa LB El Hadj Oel A Ben Hmida MA Khiari M Khalfallah T. Association of a p73 exon 2 GC/AT polymorphism with colorectal cancer risk and survival in Tunisian patients. Virchows Arch (2010) 457(3):359–68. 10.1007/s00428-010-0942-4 20644956

[288] Mzahma R Kharrat M Fetiriche F Bouasker Ben Moussa M Ben Safta Z. The relationship between telomere length and clinicopathologic characteristics in colorectal cancers among Tunisian patients. Tumour Biol (2015) 36(11):8703–13. 10.1007/s13277-015-3545-5 26047604

[289] Kassem AM El-Guendy N Tantawy M Abdelhady H El-Ghor A Abdel Wahab AH. Mutational hotspots in the mitochondrial D-loop region of cancerous and precancerous colorectal lesions in Egyptian patients. DNA Cell Biol (2011) 30(11):899–906. 10.1089/dna.2010.1186 21612400 PMC3208246

[290] Ben Sghaier R Jansen AML Bdioui A Van Wezel T Ksiaa M Elgolli L. Targeted next generation sequencing screening of Lynch syndrome in Tunisian population. Fam Cancer (2019) 18(3):343–8. 10.1007/s10689-019-00130-y 31114938

[291] Islami F Stoklosa M Drope J Jemal A. Global and regional patterns of tobacco smoking and tobacco control policies. Eur Urol Focus (2015) 1(1):3–16. 10.1016/j.euf.2014.10.001 28723352

[292] Dhieb D Belguith I Capelli L Chiadini E Canale M Bravaccini S. Analysis of genetic alterations in tunisian patients with lung adenocarcinoma. Cells (2019) 8(6):514. 10.3390/cells8060514 PMC6627075 31141932

[293] Mraihi Z Ben Amar J Bouacha H Rammeh S Hila L. EGFR mutation status in Tunisian non-small-cell lung cancer patients evaluated by mutation-specific immunohistochemistry. BMC Pulm Med (2018) 18(1):132. 10.1186/s12890-018-0706-5 30092812 PMC6085720

[294] Arfaoui Toumi A Blel A Aloui R Zaibi H Ksentinini M Boudaya MS. Assessment of EGFR mutation status in Tunisian patients with pulmonary adenocarcinoma. Curr Res Transl Med (2018) 66(3):65–70. 10.1016/j.retram.2018.02.004 29540329

[295] Bchir S Ben Nasr H Garrouch A Ben Anes A Abbassi A Tabka Z. MMP-3 (-1171 5A/6A; Lys45Glu) variants affect serum levels of matrix metalloproteinase (MMP)-3 and correlate with severity of COPD: A study of MMP-3, MMP-7 and MMP-12 in a Tunisian population. J Gene Med (2018) 20(1):e2999. 10.1002/jgm.2999 29165854

[296] Arfaoui A Kriaa L Znaidi N Gritli S Bouacha H Zermani R. Over-expression of EGFR is closely correlated to poor prognosis in Tunisian patients with non-small cell lung adenocarcinoma. J Immunoassay Immunochem (2014) 35(3):256–68. 10.1080/15321819.2013.848813 24654822

[297] Errihani H Inrhaoun H Boukir A Kettani F Gamra L Mestari A. Frequency and type of epidermal growth factor receptor mutations in moroccan patients with lung adenocarcinoma. J Thorac Oncol (2013) 8(9):1212–4. 10.1097/JTO.0b013e31829f6b4a 23945389

[298] Kaanane H El Attar H Louahabi A Berradi H Idrissi HH Khyatti M. Targeted methods for molecular characterization of EGFR mutational profile in lung cancer Moroccan cohort. Gene (2019) 705:36–43. 10.1016/j.gene.2019.04.044 31004715

[299] Hayes VM Oosthuizen CJ Kotze MJ Marx MP Buys CH. A nonsense mutation (Arg-196-Term) in exon 6 of the human TP53 gene identified in small cell lung carcinoma. Mol Cell Probes (1996) 10(5):393–5. 10.1006/mcpr.1996.0054 8910896

[300] Ezzeldin N El-Lebedy D Darwish A El Bastawisy A Abd Elaziz SH Hassan MM. Association of genetic polymorphisms CYP2A6*2 rs1801272 and CYP2A6*9 rs28399433 with tobacco-induced lung Cancer: case-control study in an Egyptian population. BMC Cancer (2018) 18(1):525. 10.1186/s12885-018-4342-5 29724170 PMC5934827

[301] Ezzeldin N El-Lebedy D Darwish A El-Bastawisy A Hassan M Abd El-Aziz S. Genetic polymorphisms of human cytochrome P450 CYP1A1 in an Egyptian population and tobacco-induced lung cancer. Genes Environ (2017) 39:7. 10.1186/s41021-016-0066-4 28074113 PMC5219678

[302] Hussein AG Pasha HF El-Shahat HM Gad DM Toam MM. CYP1A1 gene polymorphisms and smoking status as modifier factors for lung cancer risk. Gene (2014) 541(1):26–30. 10.1016/j.gene.2014.03.003 24613751

[303] B’Chir F Pavanello S Knani J Boughattas S Arnaud MJ Saguem S. CYP1A2 genetic polymorphisms and adenocarcinoma lung cancer risk in the Tunisian population. Life Sci (2009) 84(21-22):779–84. 10.1016/j.lfs.2009.03.008 19332078

[304] Tournel G Cauffiez C Leclerc J Billaut-Laden I Allorge D Chevalier D. CYP2F1 genetic polymorphism: identification of interethnic variations. Xenobiotica (2007) 37(12):1433–8. 10.1080/00498250701644403 17943660

[305] Pavanello S B’Chir F Pulliero A Saguem S Ben Fraj R El Aziz Hayouni A. Interaction between CYP1A2-T2467DELT polymorphism and smoking in adenocarcinoma and squamous cell carcinoma of the lung. Lung Cancer (2007) 57(3):266–72. 10.1016/j.lungcan.2007.04.004 17509724

[306] Rafrafi A Kaabachi S Kaabachi W Chahed B Amor AB Mbarik M. CCR2-64I polymorphism is associated with non-small cell lung cancer in tunisian patients. Hum Immunol (2015) 76(5):348–54. 10.1016/j.humimm.2015.03.003 25797207

[307] Kaabachi W Kaabachi S Rafrafi A Amor AB Tizaoui K Haj Sassi F. Association of vitamin D receptor FokI and ApaI polymorphisms with lung cancer risk in Tunisian population. Mol Biol Rep (2014) 41(10):6545–53. 10.1007/s11033-014-3538-2 24996287

[308] Kaabachi W ben Amor A Kaabachi S Rafrafi A Tizaoui K Hamzaoui K. Interleukin-17A and -17F genes polymorphisms in lung cancer. Cytokine (2014) 66(1):23–9. 10.1016/j.cyto.2013.12.012 24548421

[309] Kaabachi S Kaabachi W Rafrafi A Belkis H Hamzaoui K Sassi FH. Tumor necrosis factor gene polymorphisms in Tunisian patients with non-small cell lung cancer. Clin Lab (2013) 59(11-12):1389–95. 10.7754/clin.lab.2013.130106 24409675

[310] Rafrafi A Chahed B Kaabachi S Kaabachi W Maalmi H Hamzaoui K. Association of IL-8 gene polymorphisms with non small cell lung cancer in Tunisia: A case control study. Hum Immunol (2013) 74(10):1368–74. 10.1016/j.humimm.2013.06.033 23831257

[311] Haroun RA Zakhary NI Mohamed MR Abdelrahman AM Kandil EI Shalaby KA. Assessment of the prognostic value of methylation status and expression levels of FHIT, GSTP1 and p16 in non-small cell lung cancer in Egyptian patients. Asian Pac J Cancer Prev (2014) 15(10):4281–7. 10.7314/apjcp.2014.15.10.4281 24935385

[312] Hetta HF Zahran AM El-Mahdy RI Nabil EE Esmaeel HM Elkady OA. Assessment of circulating miRNA-17 and miRNA-222 expression profiles as non-invasive biomarkers in Egyptian Pptients with non-small-cell lung cancer. Asian Pac J Cancer Prev (2019) 20(6):1927–33. 10.31557/APJCP.2019.20.6.1927 PMC7021600 31244320

[313] Michaud DS. Chronic inflammation and bladder cancer. Urol Oncol (2007) 25(3):260–8. 10.1016/j.urolonc.2006.10.002 17483025

[314] Rosin MP Anwar WA Ward AJ. Inflammation, chromosomal instability, and cancer: the schistosomiasis model. Cancer Res (1994) 54(7 Suppl):1929s–33s. 8137314

[315] Shams TM Metawea M Salim EI. c-KIT positive schistosomal urinary bladder carcinoma are frequent but lack KIT gene mutations. Asian Pac J Cancer Prev (2013) 14(1):15–20. 10.7314/apjcp.2013.14.1.15 23534715

[316] Bowa K Mulele C Kachimba J Manda E Mapulanga V Mukosai S. A review of bladder cancer in Sub-Saharan Africa: A different disease, with a distinct presentation, assessment, and treatment. Ann Afr Med (2018) 17(3):99–105. 10.4103/aam.aam_48_17 30185677 PMC6126046

[317] Eissa S Ahmed MI Said H Zaghlool A El-Ahmady O. Cell cycle regulators in bladder cancer: relationship to schistosomiasis. IUBMB Life (2004) 56(9):557–64. 10.1080/15216540400013903 15590562

[318] Adenowo AF Oyinloye BE Ogunyinka BI Kappo AP. Impact of human schistosomiasis in sub-Saharan Africa. Braz J Infect Dis (2015) 19(2):196–205. 10.1016/j.bjid.2014.11.004 25636189 PMC9425372

[319] Shaw ME Elder PA Abbas A Knowles MA. Partial allelotype of schistosomiasis-associated bladder cancer. Int J Cancer (1999) 80(5):656–61. 10.1002/(sici)1097-0215(19990301)80:5<656::aid-ijc4>3.0.co;2-a 10048962

[320] Habuchi T Takahashi R Yamada H Ogawa O Kakehi Y Ogura K. Influence of cigarette smoking and schistosomiasis on p53 gene mutation in urothelial cancer. Cancer Res (1993) 53(16):3795–9. 8339293

[321] Feki-Tounsi M Khlifi R Louati I Fourati M Mhiri MN Hamza-Chaffai A. Polymorphisms in XRCC1, ERCC2, and ERCC3 DNA repair genes, CYP1A1 xenobiotic metabolism gene, and tobacco are associated with bladder cancer susceptibility in Tunisian population. Environ Sci Pollut Res Int (2017) 24(28):22476–84. 10.1007/s11356-017-9767-x 28803404

[322] Abdel-Rahman SZ Anwar WA Abdel-Aal WE Ghoneim MA Au WW. The CYP2D6 extensive metabolizer genotype is associated with increased risk for bladder cancer. Cancer Lett (1997) 119(1):115–22. 10.1016/s0304-3835(97)00265-6 18372530

[323] Ouerhani S Marrakchi R Bouhaha R Ben Slama MR Sfaxi M Ayed M. The role of CYP2D6*4 variant in bladder cancer susceptibility in Tunisian patients. Bull Cancer (2008) 95(2):E1–4. 10.1684/bdc.2008.0583 18304900

[324] Hadami K Dakka N Bensaid M El Ahanidi H Ameur A Chahdi H. Evaluation of glutathione S-transferase pi 1 expression and gene promoter methylation in Moroccan patients with urothelial bladder cancer. Mol Genet Genomic Med (2018) 6(5):819–27. 10.1002/mgg3.449 PMC6160697 30043549

[325] Goerlitz D El Daly M Abdel-Hamid M Saleh DA Goldman L El Kafrawy S. GSTM1, GSTT1 null variants, and GPX1 single nucleotide polymorphism are not associated with bladder cancer risk in Egypt. Cancer Epidemiol Biomarkers Prev (2011) 20(7):1552–4. 10.1158/1055-9965.EPI-10-1306 PMC3136366 21586620

[326] El Nouby KA Abd El Hameed AH Negm OE Hamouda HE El Gamal OM Ismail GM. Genetic polymorphism of glutathione-S-transferase (GST-M1 and GST-T1) in schistosomiasis -associated bladder cancer in Egyptian patients. J Egypt Soc Parasitol (2008) 38(3):991–1006. 19209780

[327] Abd El Hameed AH Negm OE El-Gamal OM Hamouda HE El Nouby KA Ismail GM. Genetic polymorphism of glutathione S-transferases M1 and T1 in Egyptian patients with bilharzial bladder cancer. Urol Oncol (2010) 28(3):296–301. 10.1016/j.urolonc.2008.09.015 19117770

[328] Ouerhani S Tebourski F Slama MR Marrakchi R Rabeh M Hassine LB. The role of glutathione transferases M1 and T1 in individual susceptibility to bladder cancer in a Tunisian population. Ann Hum Biol (2006) 33(5-6):529–35. 10.1080/03014460600907517 17381051

[329] El Desoky ES AbdelSalam YM Salama RH El Akkad MA Atanasova S von Ahsen N. NAT2*5/*5 genotype (341T>C) is a potential risk factor for schistosomiasis-associated bladder cancer in Egyptians. Ther Drug Monit (2005) 27(3):297–304. 10.1097/01.ftd.0000164197.95494.aa 15905799

[330] Anwar WA Abdel-Rahman SZ El-Zein RA Mostafa HM Au WW. Genetic polymorphism of GSTM1, CYP2E1 and CYP2D6 in Egyptian bladder cancer patients. Carcinogenesis (1996) 17(9):1923–9. 10.1093/carcin/17.9.1923 8824515

[331] Rouissi K Ouerhani S Hamrita B Bougatef K Marrakchi R Cherif M. Smoking and polymorphisms in xenobiotic metabolism and DNA repair genes are additive risk factors affecting bladder cancer in Northern Tunisia. Pathol Oncol Res (2011) 17(4):879–86. 10.1007/s12253-011-9398-3 21647780

[332] Abd El-Aal AA Bayoumy IR Basyoni MM Abd El-Aal AA Emran AM Abd El-Tawab MS. Genomic instability in complicated and uncomplicated Egyptian schistosomiasis haematobium patients. Mol Cytogenet (2015) 8(1):1. 10.1186/s13039-014-0104-5 25628757 PMC4307227

[333] Khaled HM Aly MS Mokhtar N. Chromosomal aberrations in Cis and Ta bilharzial bladder cancer: a theory of pathogenesis. Urol Oncol (2004) 22(6):443–7. 10.1016/j.urolonc.2004.07.015 15610858

[334] Fadl-Elmula I Kytola S Leithy ME Abdel-Hameed M Mandahl N Elagib A. Chromosomal aberrations in benign and malignant bilharzia-associated bladder lesions analyzed by comparative genomic hybridization. BMC Cancer (2002) 2:5. 10.1186/1471-2407-2-5 11914143 PMC101388

[335] Aly MS Khaled HM. Chromosomal aberrations in early-stage bilharzial bladder cancer. Cancer Genet Cytogenet (2002) 132(1):41–5. 10.1016/s0165-4608(01)00527-1 11801307

[336] Khaled HM Aly MS Magrath IT. Loss of Y chromosome in bilharzial bladder cancer. Cancer Genet Cytogenet (2000) 117(1):32–6. 10.1016/s0165-4608(99)00126-0 10700863

[337] Gonzalez-Zulueta M Shibata A Ohneseit PF Spruck CH,3 Busch C Shamaa M. High frequency of chromosome 9p allelic loss and CDKN2 tumor suppressor gene alterations in squamous cell carcinoma of the bladder. J Natl Cancer Inst (1995) 87(18):1383–93. 10.1093/jnci/87.18.1383 7658499

[338] Abdel Wahab AH Abo-Zeid HI El-Husseini MI Ismail M El-Khor AM. Role of loss of heterozygosity on chromosomes 8 and 9 in the development and progression of cancer bladder. J Egypt Natl Canc Inst (2005) 17(4):260–9. 17102820

[339] Khaled HM Bahnassi AA Zekri AR Kassem HA Mokhtar N. Correlation between p53 mutations and HPV in bilharzial bladder cancer. Urol Oncol (2003) 21(5):334–41. 10.1016/s1078-1439(03)00014-0 14670539

[340] Weintraub M Khaled H Zekri A Bahnasi A Eissa S Venzon D. P53 mutations in egyptian bladder-cancer. Int J Oncol (1995) 7(6):1269–74. 10.3892/ijo.7.6.1269 21552959

[341] Warren W Biggs PJ el-Baz M Ghoneim MA Stratton MR Venitt S. Mutations in the p53 gene in schistosomal bladder cancer: a study of 92 tumours from Egyptian patients and a comparison between mutational spectra from schistosomal and non-schistosomal urothelial tumours. Carcinogenesis (1995) 16(5):1181–9. 10.1093/carcin/16.5.1181 7767983

[342] Ouerhani S Rouissi K Kourda N Marrakchi R Bougatef K Riadh Ben Slama M. Combined analysis of smoking, TP53, and FGFR3 mutations in Tunisian patients with invasive and superficial high-grade bladder tumors. Cancer Invest (2009) 27(10):998–1007. 10.3109/07357900902849707 19909015

[343] Tamimi Y Bringuier PP Smit F van Bokhoven A Abbas A Debruyne FM. Homozygous deletions of p16(INK4) occur frequently in bilharziasis-associated bladder cancer. Int J Cancer (1996) 68(2):183–7. 10.1002/(sici)1097-0215(19961009)68:2<183::Aid-ijc7>3.0.Co;2-u 8900425

[344] Gutierrez MI Siraj AK Khaled H Koon N El-Rifai W Bhatia K. CpG island methylation in Schistosoma- and non-Schistosoma-associated bladder cancer. Mod Pathol (2004) 17(10):1268–74. 10.1038/modpathol.3800177 15154012

[345] Motawi TK Rizk SM Ibrahim TM Ibrahim IA. Circulating microRNAs, miR-92a, miR-100 and miR-143, as non-invasive biomarkers for bladder cancer diagnosis. Cell Biochem Funct (2016) 34(3):142–8. 10.1002/cbf.3171 26916216

[346] Zhong X Isharwal S Naples JM Shiff C Veltri RW Shao C. Hypermethylation of genes detected in urine from Ghanaian adults with bladder pathology associated with Schistosoma haematobium infection. PloS One (2013) 8(3):e59089. 10.1371/journal.pone.0059089 23527093 PMC3601097

[347] Hameed DA Yassa HA Agban MN Hanna RT Elderwy AM Zwaita MA. Genetic aberrations of the K-ras proto-oncogene in bladder cancer in relation to pesticide exposure. Environ Sci Pollut Res Int (2018) 25(22):21535–42. 10.1007/s11356-018-1840-6 29644616

[348] Ben Fradj MK Kallel A Gargouri MM Chehida MA Sallemi A Ouanes Y. Association of FokI polymorphism of vitamin D receptor with urothelial bladder cancer in Tunisians: role of tobacco smoking and plasma vitamin D concentration. Tumour Biol (2016) 37(5):6197–203. 10.1007/s13277-015-4496-6 26615419

[349] Edreis A Mohamed MA Mohamed NS Siddig EE. Molecular Detection of Epstein - Barr virus in Nasopharyngeal Carcinoma among Sudanese population. Infect Agent Cancer (2016) 11:55. 10.1186/s13027-016-0104-7 27833652 PMC5101655

[350] Ayadi W Feki L Khabir A Boudawara T Ghorbel A Charfeddine I. Polymorphism analysis of Epstein-Barr virus isolates of nasopharyngeal carcinoma biopsies from Tunisian patients. Virus Genes (2007) 34(2):137–45. 10.1007/s11262-006-0051-2 17216568

[351] Bahnassy AA Zekri AR Asaad N El-Houssini S Khalid HM Sedky LM. Epstein-Barr viral infection in extranodal lymphoma of the head and neck: correlation with prognosis and response to treatment. Histopathology (2006) 48(5):516–28. 10.1111/j.1365-2559.2006.02377.x 16623777

[352] Malik MO Banatvala J Hutt MS Abu-Sin AY Hidaytallah A El-Had AE. Epstein-Barr virus antibodies in Sudanese patients with nasopharyngeal carcinoma: a preliminary report. J Natl Cancer Inst (1979) 62(2):221–4. 216831

[353] Hadhri-Guiga B Khabir AM Mokdad-Gargouri R Ghorbel AM Drira M Daoud J. Various 30 and 69 bp deletion variants of the Epstein-Barr virus LMP1 may arise by homologous recombination in nasopharyngeal carcinoma of Tunisian patients. Virus Res (2006) 115(1):24–30. 10.1016/j.virusres.2005.07.002 16154221

[354] Janse van Rensburg E van Heerden WF Robson BA Swart TJ Engelbrecht S. Epstein-Barr virus strain characterisation in South African patients with nasopharyngeal carcinomas. Anticancer Res (2000) 20(3b):1953–7. 10928133

[355] Lazarus P Idris AM Kim J Calcagnotto A Hoffmann D. p53 mutations in head and neck squamous cell carcinomas from Sudanese snuff (toombak) users. Cancer Detect Prev (1996) 20(4):270–8. 8818386

[356] Ibrahim SO Vasstrand EN Johannessen AC Idris AM Magnusson B Nilsen R. Mutations of the p53 gene in oral squamous-cell carcinomas from Sudanese dippers of nitrosamine-rich toombak and non-snuff-dippers from the Sudan and Scandinavia. Int J Cancer (1999) 81(4):527–34. 10.1002/(sici)1097-0215(19990517)81:4<527::aid-ijc4>3.0.co;2-2 10225439

[357] Bahnassy AA Zekri AR Abdallah S El-Shehaby AM Sherif GM. Human papillomavirus infection in Egyptian esophageal carcinoma: correlation with p53, p21, mdm2, C-erbB2 and impact on survival. Pathol Int (2005) 55(2):53–62. 10.1111/j.1440-1827.2005.01804.x 15693850

[358] Makni L Ben Hamda C Al-ansari A Souiai O Gazouani E Mezlini A. Association of common IL-10 promoter gene variants with the susceptibility to head and neck cancer in Tunisia. Turk J Med Sci (2019) 49(1):123–8. 10.3906/sag-1805-21 PMC7350874 30762321

[359] Khlifi R Chakroun A Hamza-Chaffai A Rebai A. Association of CYP1A1 and CYP2D6 gene polymorphisms with head and neck cancer in Tunisian patients. Mol Biol Rep (2014) 41(4):2591–600. 10.1007/s11033-014-3117-6 24449363

[360] Gara S Abdennebi M Chatti S Touati S Ladgham A Guemira F. Association of NAT2 gene substitution mutation T341C with increased risk for head and neck cancer in Tunisia. Acta Oncol (2007) 46(6):834–7. 10.1080/02841860601096833 17653908

[361] Bougacha-Elleuch N Rebai A Mnif M Makni H Bellassouad M Jouida J. Analysis of MHC genes in a Tunisian isolate with autoimmune thyroid diseases: implication of TNF -308 gene polymorphism. J Autoimmun (2004) 23(1):75–80. 10.1016/j.jaut.2004.03.011 15236755

[362] Naidoo R Tarin M Reddi A Chetty R. Allelic imbalance and microsatellite instability in chromosomes 2p, 3p, 5q, and 18q in esophageal squamous carcinoma in patients from South Africa. Diagn Mol Pathol (1999) 8(3):131–7. 10.1097/00019606-199909000-00005 10565684

[363] Trimeche M Braham H Ziadi S Amara K Hachana M Korbi S. Investigation of allelic imbalances on chromosome 3p in nasopharyngeal carcinoma in Tunisia: high frequency of microsatellite instability in patients with early-onset of the disease. Oral Oncol (2008) 44(8):775–83. 10.1016/j.oraloncology.2007.10.001 18206419

[364] Abou-Elhamd KE Habib TN. The role of chromosomal aberrations in premalignant and malignant lesions in head and neck squamous cell carcinoma. Eur Arch Otorhinolaryngol (2008) 265(2):203–7. 10.1007/s00405-007-0420-z 17701417

[365] Toure S Mbaye F Gueye MD Fall M Dem A Lamy JB. Somatic mitochondrial mutations in oral cavity cancers among senegalese patients. Asian Pac J Cancer Prev (2019) 20(7):2203–8. 10.31557/apjcp.2019.20.7.2203 PMC6745208 31350985

[366] Phelps HM Pierce JM Murphy AJ Correa H Qian J Massion PP. FXR1 expression domain in Wilms tumor. J Pediatr Surg (2019) 54(6):1198–205. 10.1016/j.jpedsurg.2019.02.030 PMC6545243 30894247

[367] Lovvorn HN,3 Pierce J Libes J Li B Wei Q Correa H. Genetic and chromosomal alterations in Kenyan Wilms Tumor. Genes Chromosomes Cancer (2015) 54(11):702–15. 10.1002/gcc.22281 PMC4567398 26274016

[368] Gohar MK Ammar MG Alnagar AA Abd-ElAziz HA. Serum IgE and Allergy Related Genotypes of IL-4R alpha and IL-13 Genes: Association with Glioma Susceptibility and Glioblastoma Prognosis. Egypt J Immunol (2018) 25(1):19–33. 30242995

[369] Senhaji N Louati S Chbani L El Fatemi H Hammas N Mikou K. EGFR amplification and IDH mutations in glioblastoma patients of the Northeast of Morocco. BioMed Res Int (2017) 2017:8045859. 10.1155/2017/8045859 28785587 PMC5530437

[370] Taha H Yehia M Mahmoud M El-Beltagy M Ghabriel M El-Naggar S. Incidence of kiaa1549-braf fusion gene in Egyptian pediatric low grade glioma. Clin Transl Med (2015) 4:10. 10.1186/s40169-015-0052-7 25883769 PMC4392037

[371] Tarassishin L Casper D Lee SC. Aberrant expression of interleukin-1beta and inflammasome activation in human malignant gliomas. PloS One (2014) 9(7):e103432. 10.1371/journal.pone.0103432 25054228 PMC4108401

[372] Tawdy MH Abd El Nasser MM Abd El Shafy SS Nada MA El Sirafy MN Magd AH. Role of serum TRAIL level and TRAIL apoptosis gene expression in multiple sclerosis and relation to brain atrophy. J Clin Neurosci (2014) 21(9):1606–11. 10.1016/j.jocn.2013.11.056 24913933

[373] Badr El-Din NK Settin A Ali N Abdel-Hady el SK Salem FK. Cytokine gene polymorphisms in egyptian cases with brain tumors. J Egypt Natl Canc Inst (2009) 21(2):101–6. 21057561

[374] Settin A Ali N Salem FK. Cytokine gene polymorphisms in Egyptian cases with brain tumors. Egypt J Immunol (2008) 15(2):15–23. 20306684

[375] Benenemissi IH Sifi K Sahli LK Semmam O Abadi N Satta D. Angiotensin-converting enzyme insertion/deletion gene polymorphisms and the risk of glioma in an Algerian population. Pan Afr Med J (2019) 32:197. 10.11604/pamj.2019.32.197.15129 31312309 PMC6620085

[376] Hilmani S Abidi O Benrahma H Karkouri M Sahraoui S El Azhari A. Clinicopathological features and molecular analysis of primary glioblastomas in Moroccan patients. J Mol Neurosci (2013) 49(3):567–73. 10.1007/s12031-012-9868-4 22865003

[377] Senhaji N Louati S Chbani L Bardai SE Mikou K Maaroufi M. Prevalence of IDH1/2 mutations in different subtypes of glioma in the North-East population of Morocco. Asian Pac J Cancer Prev (2016) 17(5):2649–53. 27268645

[378] Smaili W Doubaj Y Laarabi FZ Lyahyai J Kerbout M Mikdame M. CALR gene mutational profile in myeloproliferative neoplasms with non-mutated JAK2 in Moroccan patients: A case series and germline in-frame deletion. Curr Res Transl Med (2017) 65(1):15–9. 10.1016/j.retram.2016.08.002 28340692

[379] Abidi O Knari S Sefri H Charif M Senechal A Hamel C. Mutational analysis of the RB1 gene in Moroccan patients with retinoblastoma. Mol Vis (2011) 17:3541–7. PMC3250372 22219649

[380] Ayari-Jeridi H Moran K Chebbi A Bouguila H Abbes I Charradi K. Mutation spectrum of RB1 gene in unilateral retinoblastoma cases from Tunisia and correlations with clinical features. PloS One (2015) 10(1):e0116615. 10.1371/journal.pone.0116615 25602518 PMC4300092

[381] Boubekeur A Louhibi L Mahmoudi K Boudjema A Mehtar N. [Molecular study of retinoblastoma in the Algerian population. Screening of Rb gene in constitutional and tumoral level]. Bull Cancer (2012) 99(2):127–35. 10.1684/bdc.2011.1529 22265791

[382] Mohammed AM Kamel AK Hammad SA Afifi HH El Sanabary Z El Din ME. Constitutional retinoblastoma gene deletion in Egyptian patients. World J Pediatr (2009) 5(3):222–5. 10.1007/s12519-009-0042-1 19693468

[383] Victor T Du Toit R Jordaan AM Bester AJ van Helden PD. No evidence for point mutations in codons 12, 13, and 61 of the ras gene in a high-incidence area for esophageal and gastric cancers. Cancer Res (1990) 50(16):4911–4. 2199031

[384] Sabry D Ahmed R Abdalla S Fathy W Eldemery A Elamir A. Braf, Kras and Helicobacter pylori epigenetic changes-associated chronic gastritis in Egyptian patients with and without gastric cancer. World J Microbiol Biotechnol (2016) 32(6):92. 10.1007/s11274-016-2048-x 27116958

[385] Buffart TE Louw M van Grieken NC Tijssen M Carvalho B Ylstra B. Gastric cancers of Western European and African patients show different patterns of genomic instability. BMC Med Genomics (2011) 4:7. 10.1186/1755-8794-4-7 21226972 PMC3033789

[386] Chetty R Naidoo R Tarin M Sitti C. Chromosome 2p, 3p, 5q and 18q status in sporadic gastric cancer. Pathology (2002) 34(3):275–81. 10.1080/00313020220131354 12109791

[387] Tu C Zeng Z Qi P Li X Yu Z Guo C. Genome-Wide analysis of 18 epstein-barr viruses isolated from primary nasopharyngeal carcinoma biopsy specimens. J Virol (2017) 91(17):e00301-17. 10.1128/JVI.00301-17 PMC5553176 28637758

[388] Gouveia MH Bergen AW Borda V Nunes K Leal TP Ogwang MD. Genetic signatures of gene flow and malaria-driven natural selection in sub-Saharan populations of the “endemic Burkitt Lymphoma belt”. PloS Genet (2019) 15(3):e1008027. 10.1371/journal.pgen.1008027 30849090 PMC6426263

[389] Simbiri KO Smith NA Otieno R Wohlford EE Daud II Odada SP. Epstein-Barr virus genetic variation in lymphoblastoid cell lines derived from Kenyan pediatric population. PloS One (2015) 10(5):e0125420. 10.1371/journal.pone.0125420 25933165 PMC4416826

[390] Geser A Lenoir GM Anvret M Bornkamm G Klein G Williams EH. Epstein-Barr virus markers in a series of Burkitt’s lymphomas from the West Nile District, Uganda. Eur J Cancer Clin Oncol (1983) 19(10):1393–404. 10.1016/0277-5379(93)90009-t 6315443

[391] Shiramizu B Barriga F Neequaye J Jafri A Dalla-Favera R Neri A. Patterns of chromosomal breakpoint locations in Burkitt’s lymphoma: relevance to geography and Epstein-Barr virus association. Blood (1991) 77(7):1516–26. 10.1182/blood.V77.7.1516.bloodjournal7771516 1849033

[392] Essop MF Engel M Close P Sinclair-Smith C Pallesen G. Epstein-barr virus in Hodgkin’s disease: frequency of a 30-bp deletion in the latent membrane protein (LMP-1) oncogene in South African patients. Int J Cancer (1999) 84(4):449–51. 10.1002/(sici)1097-0215(19990820)84:4<449::aid-ijc21>3.0.co;2-9 10404102

[393] Griffin BE. Epstein-Barr virus (EBV) and human disease: facts, opinions and problems. Mutat Res (2000) 462(2-3):395–405. 10.1016/s1383-5742(00)00028-4 11523539

[394] Farawela H Khorshied M Shaheen I Gouda H Nasef A Abulata N. The association between hepatitis C virus infection, genetic polymorphisms of oxidative stress genes and B-cell non-Hodgkin’s lymphoma risk in Egypt. Infect Genet Evol (2012) 12(6):1189–94. 10.1016/j.meegid.2012.04.007 22522002

[395] Hamadou WS Besbes S Bourdon V Youssef YB Laatiri MA Noguchi T. Mutational analysis of TP53 gene in Tunisian familial hematological malignancies and sporadic acute leukemia cases. Fam Cancer (2017) 16(1):153–7. 10.1007/s10689-016-9931-3 27619989

[396] Bhatia KG Gutierrez MI Huppi K Siwarski D Magrath IT. The pattern of p53 mutations in Burkitt’s lymphoma differs from that of solid tumors. Cancer Res (1992) 52(15):4273–6. 1638540

[397] Hosny G Farahat N Hainaut P. TP53 mutations in circulating free DNA from Egyptian patients with non-Hodgkin’s lymphoma. Cancer Lett (2009) 275(2):234–9. 10.1016/j.canlet.2008.10.029 19046801

[398] Hamdy MSA El-Saadany ZA Makhlouf MM Salama AI Ibrahim NS Gad AA. TAp73 and DeltaNp73 relative expression in Egyptian patients with lymphoid neoplasms. Tumori (2017) 103(3):268–71. 10.5301/tj.5000506 27103208

[399] Serag El-Dien MM Abdou AG Asaad NY Abd El-Wahed MM Kora M. Intratumoral FOXP3+ regulatory T cells in diffuse large B-cell lymphoma. Appl Immunohistochem Mol Morphol (2017) 25(8):534–42. 10.1097/PAI.0000000000000335 26862953

[400] Tawfeek GA Alhassanin S. HLA-G gene polymorphism in Egyptian patients with non-hodgkin lymphoma and its clinical outcome. Immunol Invest (2018) 47(3):315–25. 10.1080/08820139.2018.1430826 29388862

[401] Bel Hadj Jrad B Chatti A Laatiri A Ahmed SB Romdhane A Ajimi S. Tumor necrosis factor promoter gene polymorphism associated with increased susceptibility to non-Hodgkin’s lymphomas. Eur J Haematol (2007) 78(2):117–22. 10.1111/j.1600-0609.2006.00784.x 17087739

[402] Ibrahim A Abdel Rahman H Khorshied M Sami R Nasr N Khorshid O. Tumor necrosis factor alpha-308 and Lymphotoxin alpha+252 genetic polymorphisms and the susceptibility to non-Hodgkin lymphoma in Egypt. Leuk Res (2012) 36(6):694–8. 10.1016/j.leukres.2011.11.016 22177457

[403] Galleze A Raache R Amroun H Cherif N Fadli M Mecabih F. HLA polymorphism in Algerian children with lymphomas. J Pediatr Hematol Oncol (2015) 37(8):e458–61. 10.1097/MPH.0000000000000419 26334430

[404] El-Rashedi FH El-Hawy MA El-Hefnawy SM Mohammed MM. HFE gene mutation and iron overload in Egyptian pediatric acute lymphoblastic leukemia survivors: a single-center study. Hematology (2017) 22(7):398–404. 10.1080/10245332.2017.1289324 28211293

[405] Sofan MA Elmasry S Salem DA Bazid MM. NPM1 gene mutation in Egyptian patients with cytogenetically normal acute myeloid leukemia. Clin Lab (2014) 60(11):1813–22. 10.7754/clin.lab.2014.140121 25648021

[406] Zidan MA Kamal Shaaban HM Elghannam DM. Prognostic impact of Wilms tumor gene mutations in Egyptian patients with acute myeloid leukemia with normal karyotype. Hematology (2014) 19(5):267–74. 10.1179/1607845413Y.0000000129 24074521

[407] Ouerhani S Gharbi H Menif S Safra I Douzi K Abbes S. KIT mutation detection in Tunisian patients with newly diagnosed myelogenous leukemia: prevalence and prognostic significance. Cancer Genet (2012) 205(9):436–41. 10.1016/j.cancergen.2012.05.008 22939396

[408] Elghannam DM Abousamra NK Shahin DA Goda EF Azzam H Azmy E. Prognostic implication of N-RAS gene mutations in Egyptian adult acute myeloid leukemia. Egypt J Immunol (2009) 16(1):9–15. 20726318

[409] Al-Tonbary Y Mansour AK Ghazy H Elghannam DM Abd-Elghaffar HA. Prognostic significance of foetal-like tyrosine kinase 3 mutation in Egyptian children with acute leukaemia. Int J Lab Hematol (2009) 31(3):320–6. 10.1111/j.1751-553X.2008.01039.x 18336585

[410] Durosinmi MA Faluyi JO Ogunsanwo BA. Chromosomal aberrations in Nigerians with haematological malignancies: preliminary report. Afr J Med Med Sci (1993) 22(3):21–7. 7839908

[411] Anyanwu NCJ Ella EE Aminu M Kazeem HM. Detection of NRAS G12D and NRAS G13C mutant genes among apparently healthy and haematologic malignant individuals in Federal Capital Territory, Nigeria. J Immunoassay Immunochem (2019) 40(6):605–16. 10.1080/15321819.2019.1668407 31538838

[412] Hagiwara N Berry-Bobovski L Francis C Ramsey L Chapman RA Albrecht TL. Unexpected findings in the exploration of African American underrepresentation in biospecimen collection and biobanks. J Cancer Educ (2014) 29(3):580–7. 10.1007/s13187-013-0586-6 PMC4026340 24243440

[413] Durvasula A Sankararaman S. Recovering signals of ghost archaic introgression in African populations. Sci Adv (2020) 6(7):eaax5097. 10.1126/sciadv.aax5097 32095519 PMC7015685

[414] Odedina FT Dagne G LaRose-Pierre M Scrivens J Emanuel F Adams A. Within-group differences between native-born and foreign-born Black men on prostate cancer risk reduction and early detection practices. J Immigr Minor Health (2011) 13(6):996–1004. 10.1007/s10903-011-9471-8 21547350

[415] Sherman RM Forman J Antonescu V Puiu D Daya M Rafaels N. Assembly of a pan-genome from deep sequencing of 910 humans of African descent. Nat Genet (2019) 51(1):30–5. 10.1038/s41588-018-0273-y PMC6309586 30455414

